# Improved Muscle Function in Duchenne Muscular Dystrophy through L-Arginine and Metformin: An Investigator-Initiated, Open-Label, Single-Center, Proof-Of-Concept-Study

**DOI:** 10.1371/journal.pone.0147634

**Published:** 2016-01-22

**Authors:** Patricia Hafner, Ulrike Bonati, Beat Erne, Maurice Schmid, Daniela Rubino, Urs Pohlman, Thomas Peters, Erich Rutz, Stephan Frank, Cornelia Neuhaus, Stefanie Deuster, Monika Gloor, Oliver Bieri, Arne Fischmann, Michael Sinnreich, Nuri Gueven, Dirk Fischer

**Affiliations:** 1 Division of Neuropaediatrics, University of Basel Children's Hospital, Basel, Switzerland; 2 Department of Neurology, University of Basel Hospital, Basel, Switzerland; 3 Department of Biomedicine, University of Basel, Basel, Switzerland; 4 Interdisciplinary Center of Nutritional and Metabolic Diseases, St. Claraspital, Basel, Basel, Switzerland; 5 Paediatric Orthopaedic Department, University of Basel Children's Hospital, Basel, Switzerland; 6 Division of Neuropathology, Institute of Pathology, University of Basel Hospital, Basel, Switzerland; 7 Therapy Department, University of Basel Children's Hospital, Basel, Switzerland; 8 Hospital Pharmacy, University of Basel Hospital, Basel, Switzerland; 9 Department of Radiology, Division of Radiological Physics, University of Basel Hospital, Basel, Switzerland; 10 Division of Neuroradiology, University of Basel Hospital, Basel, Switzerland; 11 Pharmacy, School of Medicine, University of Tasmania, Hobart, TAS, Australia; University of Sevilla, SPAIN

## Abstract

Altered neuronal nitric oxide synthase function in Duchenne muscular dystrophy leads to impaired mitochondrial function which is thought to be one cause of muscle damage in this disease. The study tested if increased intramuscular nitric oxide concentration can improve mitochondrial energy metabolism in Duchenne muscular dystrophy using a novel therapeutic approach through the combination of L-arginine with metformin. Five ambulatory, genetically confirmed Duchenne muscular dystrophy patients aged between 7–10 years were treated with L-arginine (3 x 2.5 g/d) and metformin (2 x 250 mg/d) for 16 weeks. Treatment effects were assessed using mitochondrial protein expression analysis in muscular biopsies, indirect calorimetry, Dual-Energy X-Ray Absorptiometry, quantitative thigh muscle MRI, and clinical scores of muscle performance. There were no serious side effects and no patient dropped out. Muscle biopsy results showed pre-treatment a significantly reduced mitochondrial protein expression and increased oxidative stress in Duchenne muscular dystrophy patients compared to controls. Post-treatment a significant elevation of proteins of the mitochondrial electron transport chain was observed as well as a reduction in oxidative stress. Treatment also decreased resting energy expenditure rates and energy substrate use shifted from carbohydrates to fatty acids. These changes were associated with improved clinical scores. In conclusion pharmacological stimulation of the nitric oxide pathway leads to improved mitochondria function and clinically a slowing of disease progression in Duchenne muscular dystrophy. This study shall lead to further development of this novel therapeutic approach into a real alternative for Duchenne muscular dystrophy patients.

**Trial Registration:**

ClinicalTrials.gov NCT02516085

## Introduction

Duchenne muscular dystrophy (DMD) is an X-linked recessive neuromuscular disorder that affects 1 in 3.500–6.000 male births. DMD is characterized by rapid and irreversible replacement of normal muscle by connective tissue and fat. Although the disease causing gene product, dystrophin, is present in many different tissues throughout the body, disease pathology is predominantly restricted to muscle tissue. In the muscle, dystrophin is located close to the inner surface of the plasmalemma and interacts as a structural protein both with a number of membrane proteins that form the dystrophin-associated glycoprotein complex (DGC), and cytoskeletal proteins[1, 2]. Therefore, loss of dystrophin in DMD is associated with loss of cytoskeletal and sarcolemmal integrity. It is believed that this structural defect gives rise to dysregulated calcium homeostasis through mechano-sensitive Ca^++^-channels, activation of proteases, such as calpain, and increased production of reactive oxygen species (ROS), which cause protein and membrane damage. One of the major sources of cellular ROS are mitochondria, implying altered mitochondrial function in DMD. However, while patients with mitochondrial dysfunction disorders frequently display impaired muscle function [3], mitochondrial dysfunction as a feature of DMD is not generally accepted despite numerous reports. One of the first publications that described impaired oxidative phosphorylation as a feature of DMD was reported in 1985 [4]. Later, using ^31^P magnetic resonance spectroscopy, increased ADP and Pi levels relative to ATP and reduced phosphocreatine levels were found in muscle of DMD patients [5]. Sperl et al. [6] also reported decreased oxidation rates in muscle biopsies from DMD patients and some indication of loose coupling of oxidative phosphorylation in mitochondria from those patients. These findings were also supported by later observations of reduced rates of cellular respiration and lower activities of enzymes of the mitochondrial respiratory chain in biopsy samples of a DMD patient.

Some of this mitochondrial dysfunction is recapitulated in the *mdx*-mouse model of DMD. Analysis of skeletal *mdx* muscle showed a 50% decrease in the activity of all respiratory chain linked enzymes compared to control animals[7]. The authors also reported that isolated mitochondria from *mdx* muscles had only 60% of maximal respiration rates compared to control and attributed this impairment to a Ca^++^-overload of dystrophin-deficient muscle fibers. Interestingly, this study identified no deficiencies in cardiac muscle. Contrary to that, Braun et al. [8] reported that irrespective of muscle type, the absence of dystrophin had no effect on the maximal capacity of oxidative phosphorylation, or on coupling between oxidation and phosphorylation. Finally, Millay et al. [9] reported a strong link between mitochondrial-dependent necrosis and muscular dystrophy in several mouse models (incl. the *mdx*-model), which strongly suggests that mitochondria play a major role in the pathology of DMD. Consistent with impaired mitochondrial function in DMD, low fat utilization as energy substrate during early stages of the disease has been suggested [10, 11]. This hypothesis is supported by observations that muscle tissue is being increasingly replaced by adipose tissue in DMD patients [12].

In DMD, loss of dystrophin also results in a severe reduction of neuronal nitric oxide (NO) synthase (nNOS) activity [13], which under normal conditions converts intramuscular L-arginine to NO [14]. NO stimulates mitochondrial biogenesis by increasing SIRT1 and PGC-1α concentrations [15], and is also critical for regulating muscular energy balance by activating AMP-activated protein kinase (AMPK) [16]. It is thought that NO and AMPK synergistically increase mitochondrial function and biogenesis through independent mechanisms. Therefore, impaired nNOS function could contribute to the observed mitochondrial dysfunction in DMD. Children with DMD have elevated synthesis of asymmetric dimethylarginine (ADMA), diminished Homoarginine (hArg) synthesis and reduced NO bioavailability compared to healthy children [17]. Increasing NO levels to stimulate mitochondrial function, to reduce oxidative stress, and to improve fat utilization for energy production appears promising to ameliorate the pathology of DMD. Skeletal muscle nNOS activation is AMPK dependent [18] and there is broad evidence for beneficial effects of AMPK activation in the mdx mouse model. 5-Aminoimidazole-4-carboxamide ribonucleotide (AICAR), an AMPK inducer, reduces muscle fatigability and improves performance of muscles from mdx mice by increasing PGC-1α and mitochondrial biogenesis [19]. Chronic AMPK stimulation triggers beneficial adaptations [20] and ameliorates the dystrophic phenotype in the mdx mouse model [21]. One of the best known pharmacologically AMPK activators is metformin that can elevate AMPK concentrations in human skeletal muscle [22]. In accordance, metformin stimulates PGC-1α expression in the mdx mouse [23] and protects skeletal muscle from toxic degeneration [24]. Taken together, there is evidence the metformin possibly could ameliorate the dystrophic phenotype via augmenting the AMPK dependent nNOS stimulation.

To test this hypothesis of a synergistic effect of NO and AMPK to stimulate mitochondrial function, this study aimed to evaluate the subclinical and clinical benefits of the combined therapy with the NO precursor L-arginine, and the pharmacological AMPK activator and indirect nNOS stimulator metformin, in DMD patients. This approach should potentiate the effect of either a solely therapy with metformin or L-Arginine.

## Material and Methods

### Ethics statement

An investigator-initiated, open-label, single-center, proof-of-concept-study approved by the local Ethics Committee (EKBB EK209/11) and National Swiss Drug Agency (Swissmedic) (2012DR2001) was conducted. Approval was obtained October 25^th^ 2011. Participant recruitment started in January 2012; last follow up visit was performed in October 2012. Patients and parents were informed on preclinical data, alternative treatments, risks, and possible benefits of the study. Oral informed assent from affected children and written informed consent from parents was obtained.

This study was registered after enrolment of participants started because registration was not mandatory at that time. The authors confirm that all ongoing and related trials for this drug/intervention are registered.

### Patients

A total of five genetically proven DMD patients were enrolled, with four of them not treated with steroids. Mean age was 7 years and 10 months (7 ± 0.75 years) at baseline. Patients were recruited from the outpatient department at the University Children’s Hospital Basel (Switzerland), as well as from the DMD patient registries from Switzerland, Germany, and Austria. Data was collected at the University Children’s Hospital in Basel, Switzerland. Inclusion criteria were molecular diagnosis of DMD, age 7 to 10 years at inclusion and independent ambulation. Exclusion criteria were intake of L-arginine or metformin within the last three months, previous participation in any other therapeutic trial for DMD, significant concomitant illness or impairment of renal, hepatic, respiratory, or cardiac function, or known hypersensitivity to study medication.

**Fig 1:**
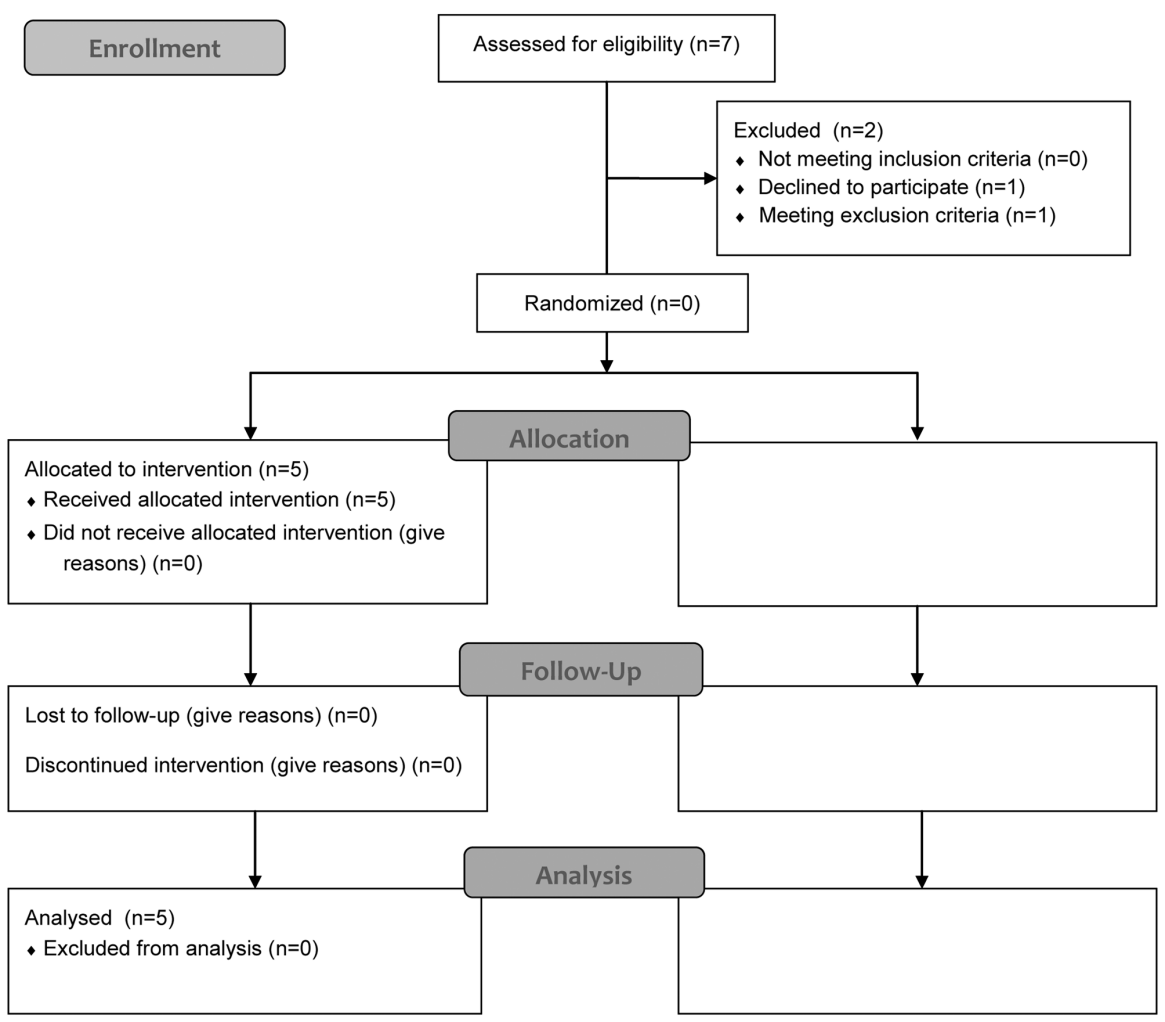
Study profile according CONSORT flow chart.

Patient number was low because of the intention of this pilot study to proof the concept of pharmacological stimulation of the nitric oxide pathway by multiple and also invasive assessments. The patient disposition of this pilot- trial is illustrated in Fig 1.

### Treatment and dose selection

L-argininehydrochloride drinking solution (L-argininehydrochloride, Selectchemie Zürich, Switzerland) was administered daily as an oral dose of 3 x 2.5g (t.i.d.) over the study period of 16 weeks. The L-arginine dose is in line with previous studies that reported a doubling of L-arginine serum concentration in children aged 7 to 17 years [25, 26].

Metformin is an oral biguanide antidiabetic drug approved by Swissmedic for insulin resistance and type 2 diabetes. Metformin-associated lactic acidosis is a very rare but relevant side effect only occurring in patients with impaired renal function. Metformin may cause mild and transient gastrointestinal side effects but no severe side effects in otherwise healthy subjects. Metformin (Sandoz Pharmaceuticals AG, Rotkreuz, Switzerland) was given twice daily at a dose of 2x250 mg (b.i.d) over the study period of 16 weeks.

Medication was administered at home by the parents. To assess compliance of medication intake empty and full vials and blisters were returned by the patients at the end of the 16 week study period.

### Study design and safety

At screening, patients fulfilling all inclusion criteria and no exclusion criteria and giving informed consent were enrolled into the study. At baseline and after completion of the trial (week 16), muscle metabolism was assessed in all patients by a punch biopsy of vastus lateralis muscle and indirect calorimetry. Furthermore, quantitative thigh muscle MRI, whole-body Dual-Energy X-Ray absorptiometry (DEXA), clinical assessments, and laboratory parameters were performed. Treatment was started one day after baseline visit. For safety reasons selected laboratory parameters and vital signs were assessed subsequently (at week 2, 4, and 8) and at the final visit at week 16. All parameters at baseline and at week 16 were performed in the same order and time schedule to minimize measuring errors.

### Clinical assessment of muscle function

Clinical response to treatment was assessed by the motor function measure (MFM), which was performed by certified physiotherapists and is a validated clinical score assessing both ambulatory and non-ambulatory patients [27]. This measure was chosen to facilitate the assessment of patients who might lose independent ambulation during the trial. The MFM is recommended in the guidelines on the clinical investigation of medicinal products for the treatment of DMD published by the European Medicines Agency [28]. In addition, timed motor performance tests (2 min walking distance, timed Gower`s maneuver and 10 meter walking time) were employed [29].

### Indirect calorimetry and DEXA

After intake of a standardized meal and a fasting period of at least 12 hours indirect calorimetry was performed (Deltatrac II, MBM-200 Metabolic Monitor) [30]. DEXA was performed to determine body’s content of bone, fat, and muscle. This technique uses two slightly diverging sources of X radiation for tissue differentiation. The irradiation dose was 1–2 μSv (Hologic QDR 4500 A (S/N 45959) [31].

### Magnetic resonance imaging (MRI)

To evaluate the muscle fat fraction (MFF) and healthy muscle fraction (HMF), a two-point Dixon method (2PD) was used [32]. MRI was performed on a 1.5 Tesla scanner (Magnetom Avanto, Siemens Healthcare, Erlangen, Germany) as described [33].

### Muscle biopsy analysis

Muscle biopsies of vastus lateralis muscle were obtained from all patients before and after treatment to assess treatment effects. Furthermore, four control muscle biopsies were used from age-matched healthy persons who underwent muscle biopsies for diagnosis of neuromuscular symptoms but ultimately were deemed to be normal by means of combined clinical, serological, electrophysiological, and histological criteria.

Tissue was frozen and stored at -80°C until processing. To assess markers of nitric oxide levels, mitochondrial biogenesis, and ROS-induced damage we measured the levels of cGMP, nitrotyrosine, carbonylated proteins, and the levels of different mitochondrial respiratory complexes by ELISA or Western blot respectively. Each analysis was repeated three times for each muscle biopsy sample. Two different protein extractions were performed: one procedure without detergent to be used for the ELISA format and a detergent-based one adapted for muscle tissues to be used in western blot detection. Protein content of extracts was measured using the Lowry based DC Protein Assay (Bio-Rad Laboratories, U.S.). For measuring the levels of cGMP in muscle extracts a commercial cGMP ELISA Kit (Cell Biolabs Inc., San Diego, Ref: STA-505) was used following the manufacturer’s instructions. The nitrotyrosine content was quantified using a colorimetric OxiSelect Nitrotyrosine ELISA Kit (Cell Biolabs Inc., San Diego, Ref: STA-305) according to the manufacturer’s recommendations. Carbonylated proteins were quantified using a commercial kit (OxiSelect Protein Carbonyl ELISA Kit;Cell Biolabs Inc., San Diego, Ref: STA-310) following the manufacturer’s manual. The optical densities of the substrate product were measured at 450nm wavelength using a SpectraMAX 190 (Molecular Devices LLC, USA) plate reader / photospectrometer and SoftMax Pro v4.8 (Molecular Devices LLC, USA) software. To detect and measure the OXPHOS complexes in the muscle extracts, samples of 20 g of protein were applied and separated on a 12% acrylamide gel, using a Mini PROTEAN 3 System from Bio-Rad Laboratories, U.S. Separated proteins were transferred on Immobilon-FL PVDF transfer membrane with a pore size of 0.45μm (Millipore Inc., Ref: IPFL00010). The blotted membranes were blocked in a TopBlock (LubioScience, Ref: TB232010, Lucerne, CH) containing solution and then incubated o/n at 4°C with the MitoProfile Total OXPHOS mouse monoclonal antibody cocktail (Abcam plc, Ref: ab110411, Cambridge, U.S.) at the manufacturer’s suggested dilution. Additionally, a rabbit-anti-GAPDH antibody (Sigma, Ref: G9545, Missouri, U.S.) at a dilution of 1:5000 and a rabbit-anti-α-Skeletal Muscle Actin (ACTA) antibody (Abcam plc, Ref: ab113417, Cambridge, U.S.) at a dilution of 1:3500 were added to the incubation solution containing TopBlock. After washing the blots in Tris-Buffered Saline and Tween 20 (TBS-T) a mix of Goat-anti-mouse IgG AlexaFluor680 coupled 2nd antibody (Molecular Probes Ref: A21057, Life Technologies, Eugene, U.S.) at a dilution of 1:7500 and Goat-anti-Rabbit IgG IRDye800 coupled 2nd antibody (Rockland Inc., Ref: 611-132-122, Gilbertsville, U.S.) at a dilution of 1:7500 were incubated for 50 min. at RT. After washing the membranes the fluorescence was scanned on an Odyssey infrared scanner (LICOR Biosciences GmbH, Bad Homburg, Germany) using the Odyssey 2.1 software configured with identical settings for all scans and measurements. The bands were automatically identified and measured by the same software using identical settings for all blots and all samples. Every sample was processed and measured in three different experiments under the same conditions. The integrated intensity of these three measurements was averaged and the mean value of three measurements was used as single value for each patient sample.

### Statistical analysis

Statistical analyses were performed using SPSS 22 (IBM, Statistical Package for the Social Sciences). For comparisons between controls and Duchenne patients the nonparametric Mann–Whitney U test was applied, while baseline and follow-up measurements were compared using the Wilcoxon signed-rank test. A level of significance alpha = 0.05 was selected. Correlations were calculated using Pearson product-moment correlation coefficient (R).

## Results

### Safety, tolerability, and laboratory testing

No severe adverse events occurred and no patient dropped out of the study. During the first week of treatment four of the five patients suffered from mild diarrhea, a known side effect of metformin, but symptoms resolved spontaneously. Slightly increased mean resting L-arginine plasma concentrations and global arginine bioavailability were detected, which did not reach significance. Repeated safety assessments showed fluctuating levels (up to + / − 50% individual changes) of individual creatine kinase and associated transaminase concentrations. No consistent changes in L-arginine related amino acids, liver and renal function tests, and markers of carbohydrate and lipid metabolism or changes in creatine kinase levels were evident. Baseline and post-treatment patient characteristics, safety, and laboratory values are shown in Table 1.

**Table 1:** Summary of basic characteristics and laboratory data.

	DMD Pretreatment		DMD Posttreatment		Change		
	Mean	(SD)	Mean	(SD)	Mean	%	*p–*value*
**Basic characteristics**							
Age, years	7.9	0.4	8.2	0.4	0.3	-	-
Height, m	1.22	0.1	1.24	0.1	0.0	2%	0.109
Weight, kg	22.7	3.0	22.8	3.1	0.2	1%	0.104
BMI, kg / m2	15.2	2.0	14.9	1.7	-0.3	-2%	0.225
**Laboratory values**							
L-arginine, μmol/l	68	25	82	52	14	20%	1.000
L-citrulline, μmol/l	29	10	24	10	-5	-18%	0.273
L-ornithine, μmol/l	65	19	70	17	5	8%	1.000
GAB	0.7	0.1	0.9	0.7	0.2	26%	0.465
Urea	5,5	0,8	5,4	0,5	-0,1	-2%	0.581
ASAT, mmol/l	297	84	345	106	48	16%	0.043
ALAT, mmol/l	475	171	582	247	107	23%	0.225
Creatinekinase, U/l	11637	4085	13082	3693	1445	12%	0.138
Creatinine, μmol/l	15.2	2.9	15.8	1.9	0.6	4%	0.317
Glucose, mmol/l	4.7	0.2	4.8	0.6	0.1	2%	0.705
Triglycerides, mmol/l	1.2	0.3	0.9	0.3	-0.3	-25%	0.109
Cholesterine, mmol/l	4.1	0.7	4.0	0.8	-0.1	-2%	0.276
HDL cholesterine, mmol/l	1.2	0.1	1.2	0.2	0.0	1%	0.892
LDL cholesterine, mmol/l	2.4	0.7	2.4	0.9	0.0	1%	0.893
Adiponectin μg/ml	7.6	4.2	7.8	5.0	0.3	3%	0.498
Leptin μg/l	0.3	0.3	0.5	0.7	0.2	80%	0.786

GAB = global arginine bioavaliability, defined as L-arginine /(L-ornithine + L-citrulline)

*P values were calculated using the Wilcoxon signed-rank test

### Muscle biopsies

**Fig 2:**
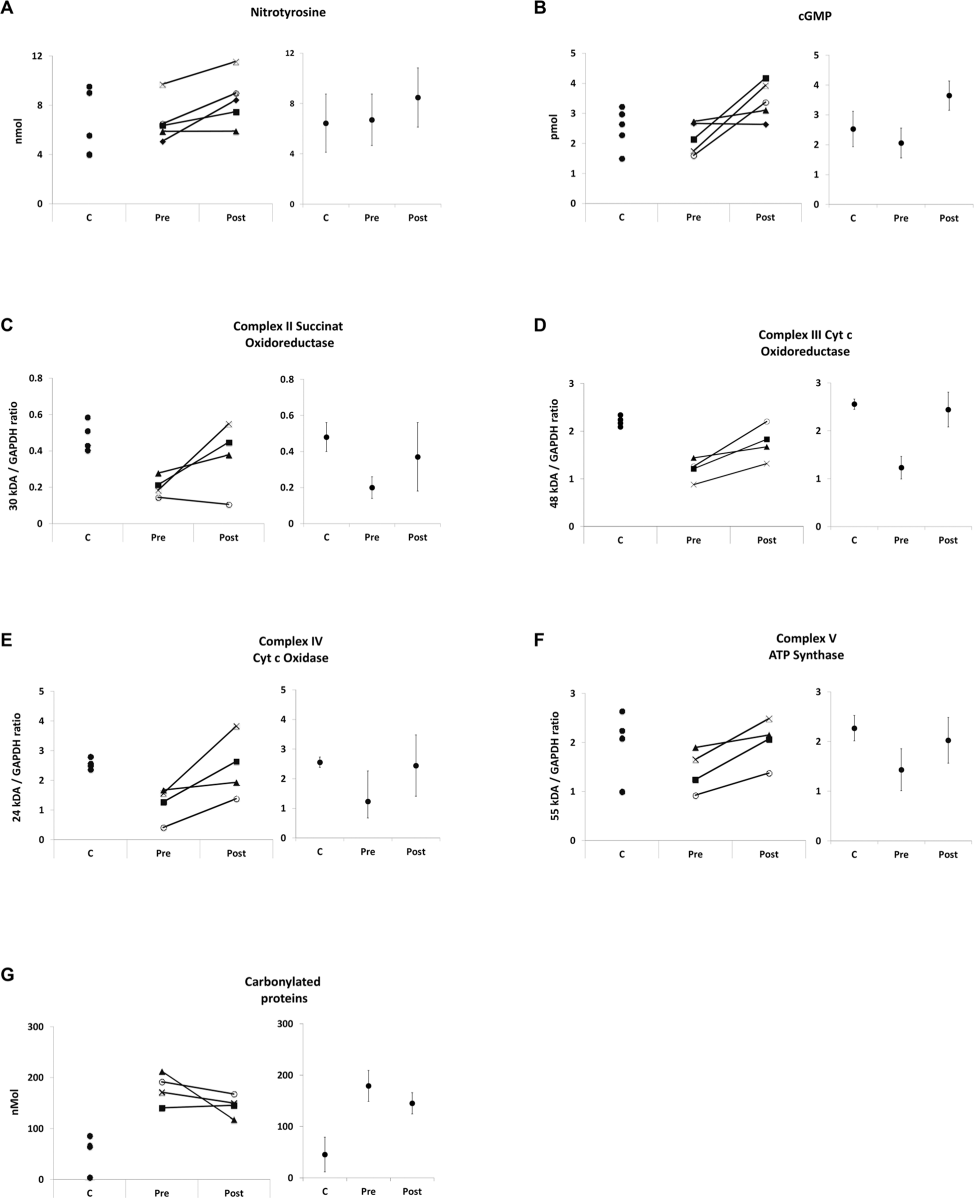
Muscle biopsy. Muscle biopsy findings, individual control (C) concentrations and individual DMD changes before (PRE) and after (POST) treatment as well as point estimates with 95% confidence intervals. A = nitrotyrosine ELISA, concentration is provided in nmol / 7.9 μg protein, B = cGMP ELISA, concentration is provided in pmol / 6.4 μg protein, C = western blot succinat oxidoreductase (complex II) / GAPDH ratio, D = western blot cytochrome c oxidoreductase (complex III) / GAPDH ratio, E = western blot cytochrome c oxidase (Complex IV) / GAPDH ratio, F = western blot ATP synthase (Complex V) / GAPDH ratio, G = carbonylated protein ELISA (nmol / 1 μg protein).

To evaluate whether treatment with arginine and metformin was able to modulate muscle NO and mitochondrial content, different markers of NO, OXPHOS, and ROS pathways were assessed in vastus lateralis muscle tissue. Protein content from patient biopsy material was in the range of 5 to 35.5 mg (mean value = 12.98 mg) while control biopsy tissues yielded 18.9 mg to 47 mg (mean value = 35.03 mg) total protein. ELISA extracts from one patient (J6 after treatment) was contaminated with Tissue-Tek© optimum cutting temperature (OCT) compound. Repeated analyses of control muscle with and without OCT contamination revealed that OCT did not interfere with the cGMP and nitrotyrosine ELISA but with carbonylated protein ELISA (not shown). Therefore, carbonyl ELISA analysis contained 4 sample pairs only. Western blot extracts of one biopsy (MA3 after treatment) did not include a sufficient amount of protein therefore OXPHOS expression could only be analyzed in four out of five sample pairs. Muscle biopsy results (mean values, standard error of the mean, changes, and *p*-values) are shown in Table 2, individual changes in Fig 2.

**Table 2:** Muscle biopsy analysis.

Pathway	Variable	Control		DMD Pretreatment		DMD Posttreatment		Difference		Change	
		Mean	(SEM)	Mean	(SEM)	Mean	(SEM)	Controls vs. DMD Pretreatment		Pretreatment vs. Posttreatment	
								%	*p*-value*	%	*p*-value**
**Nitric oxide**	**cGMP**	2.53	0.30	2.06	0.23	3.65	0.28	-19%	0.413	58%	0.068
	**Nitrotyrosin**	6.43	1.20	6.70	0.79	8.47	0.93	4%	0.548	26%	0.043
**Oxphos**	**Complex II (Succinat Oxidoreductase)**	0.48	0.04	0.20	0.02	0.37	0.09	-58%	0.029	85%	0.144
	**Complex III (Cyt c Ocidoreductase)**	2.21	0.05	1.20	0.12	1.76	0.18	-46%	0.029	47%	0.068
	**Complex IV (Cyt c Oxidase)**	2.56	0.09	1.23	0.28	2.44	0.53	-52%	0.029	99%	0.068
	**Complex V (ATP Synthase)**	2.27	0.13	1.43	0.22	2.02	0.23	-37%	0.029	41%	0.068
**ROS**	**Carbonylated Proteins**	45.29	17.19	179.10	15.32	145.34	10.52	296%	0.016	-19%	0.144

DMD = Duchenne Muscular Dystrophy; cGMP = cyclic guanosinmonophosphat; OXPHOS = oxidative phosphorylation; ROS = reactive oxygen species

*P values were calculated using the Mann-Whitney U test

**P values were calculated using the Wilcoxon signed-rank test

While at baseline, mean cGMP and nitrotyrosine concentrations did not differ significantly between DMD and control muscle (Table 2, Fig 2A and 2B), distinct impaired mitochondrial protein expression in DMD muscle was evident (Table 2, Fig 2C–2F). Mean OXPHOS protein / GAPDH ratios of complexes II, III, IV, and V were all between -37 and -58% lower in DMD (*p*< 0.05) compared to control muscles. Carbonylated proteins were four times higher compared to control samples (*p* = 0.016) demonstrating high levels of oxidative stress in DMD muscle (Table 2, Fig 2G).

After 16 weeks of treatment with L-arginine and metformin, a significant increase of mean nitrotyrosine concentrations by 26% (*p* = 0.043) and a consistent, but not significant cGMP increase of 58% (*p* = 0.068) were observed in DMD patients. In line with a role for cGMP to control mitochondrial content, a relevant increase of all OXPHOS complexes between 41 to 99% was detected after treatment, albeit the increased expression observed for complexes II (85%, *p* = 0.144), III (47%, *p* = 0.068), IV (99%, *p* = 0.068) and V (41%, *p* = 0.068) was not significant (Table 2, Fig 2D and 2F). This change of mitochondrial content was associated with a trend towards reduced levels of carbonylated proteins in DMD patients (19% reduction after treatment (*p* = 0.144)) (Table 2, Fig 2G). Individual analysis of nitrotyrosine, cGMP, OXPHOS, and ROS concentrations showed consistent responses for the non-significant changes, too.

As expected from the molecular changes observed above, cGMP levels correlated positively with the expression of mitochondrial protein complexes III (R = 0.66) and IV (R = 0.57) in DMD patients. Furthermore, cGMP concentrations after treatment showed a positive correlation with nitrotyrosine concentration (R = 0.99), and positively correlated with changes of complex III (R = 0.86) and complex IV (R = 0.92) concentrations. In DMD muscles, cGMP (R = -0.33), complex III (R = -0.46), and complex IV (R = -0.39) concentrations showed negative correlation with carbonylated proteins, indicating that cGMP-dependent increase in mitochondrial content is not associated with oxidative stress in DMD.

### Indirect calorimetry, DEXA, and quantitative muscle MRI

**Fig 3:**
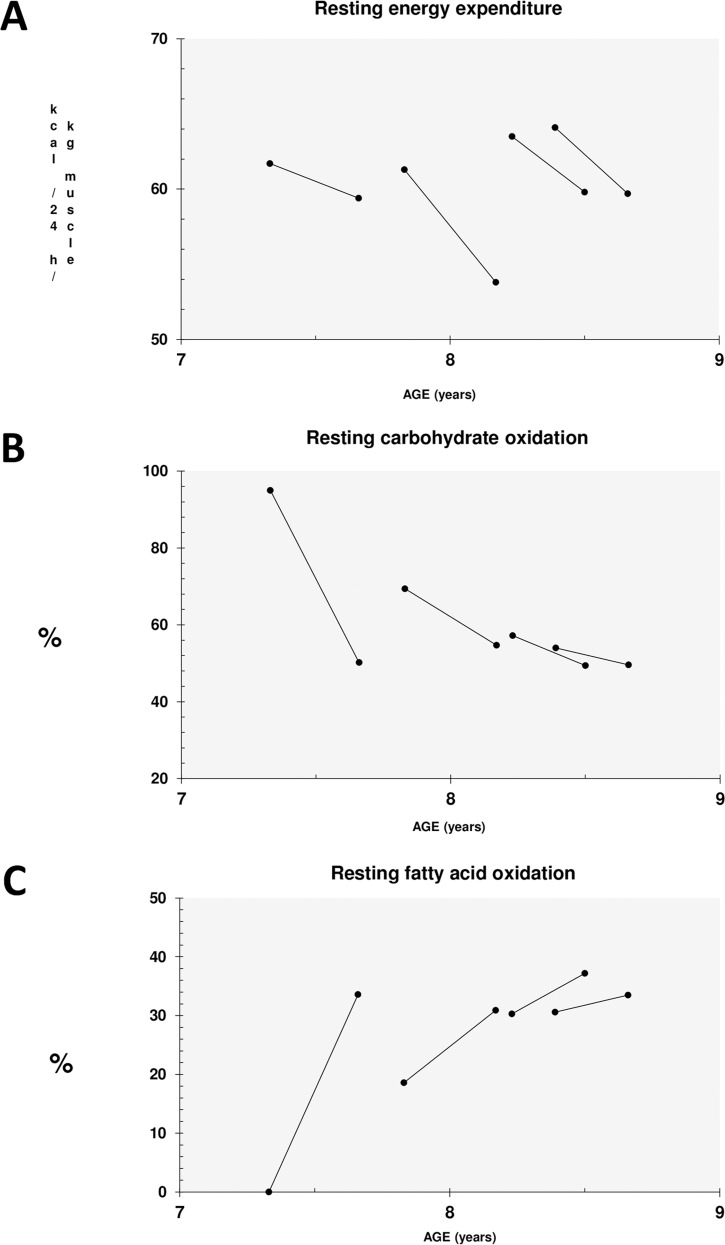
Indirect calorimetry. Indirect calorimetry results demonstrating consistently decreased rates of resting energy expenditure per kg muscle in 24 hours (A), reduced usage of carbohydrates as oxidative fuel (B), and increased usages of fatty acids (C) in oxidative energy metabolism.

To assess the metabolic consequences of stimulated mitochondrial protein expression indirect *in vivo* calorimetry was performed in DMD patients before and after treatment. Only data from four patients were analyzed before and after treatment, since one patient failed to finish the first examination (before treatment). A considerable, but statistically not significant decrease of the relative resting energy expenditure (REE) / kg muscle (mean change 7.2%, *p* = 0.068), associated with a clear change of energy substrate use (Table 3, Fig 3A) was detected after treatment. Treatment decreased the mean relative carbohydrate oxidation rate (Fig 3B) while increasing the mean relative fatty acid oxidation (Fig 3C).

It is important to point out that in line with increased consumption of fatty acids, we also did not observe a significant worsening of the muscle-to-fat ratio in treated patients. DEXA scans showed that average whole body lean content and fat content did not change significantly. Quantitative muscle MRI did not show relevant changes to the muscle fat fraction (MFF) measured by the 2PD method after 16 weeks of treatment. Though not significant, there was an unexpected trend towards increased healthy muscle area (HMA) of all thigh muscles by 6.2% (Table 3).

**Table 3:** Summary of indirect calorimetry, DEXA, and muscle MRI data.

	DMD Pretreatment		DMD Posttreatment		Change		
	Mean	(SD)	Mean	(SD)	Mean	(%)	*p* value*
**Indirect calorimetry**							
REE, kcal/24 h	1007	135	955	130	-52	-5.4%	0.068
Relative REE, kcal/24 h/kg muscle	62.7	1.4	58.2	2.9	-4.5	-7.2%	0.068
Carbohydrate oxidation, %	68.9	18.6	51.0	2.5	-17.9%		0.068
Carbohydrate oxidation, kcal/kg muscle	10.2	2.6	7.1	0.1	-3.2	-31.1%	0.068
Fatty acid oxidation, %	19.9	14.4	33.8	2.6	13.9%		0.109
Fatty acid oxidation, kcal/kg muscle	1.3	1.0	2.1	0.2	0.8	61.5%	0.109
**DEXA scan, body composition**							
Lean Mass, kg	16.1	1.9	16.4	2.4	0.3	1.8%	0.500
Lean Mass of whole body, %	71.3	2.4	71.4	3.3	0.1%		0.786
Fat, kg	5.8	1.2	5.82	1.3	0.02	0.3%	0.893
Fat of whole body, %	25.5	2.7	25.2	3.7	-0.3%		0.686
**MRI**							
**Muscle fat fraction (%)**							
Quadriceps	11.9	5.8	13.2	6.6	1.3%		0.080
all thigh muscles	14.0	5.7	15.5	6.8	1.5%		0.080
**Healthy muscle area (mm2)**							
Quadriceps	4149	1331	4352	1686	203	4.9%	0.500
all thigh muscles	8106	2201	8611	2628	505	6.2%	0.345

REE = Resting Energy Expenditure; DEXA = Dual-Energy X-Ray absorptiometry; 2PD = two point Dixon method; MFF = muscle fat fraction

*P values were calculated using the Wilcoxon signed-rank test

### Clinical scores

**Fig 4:**
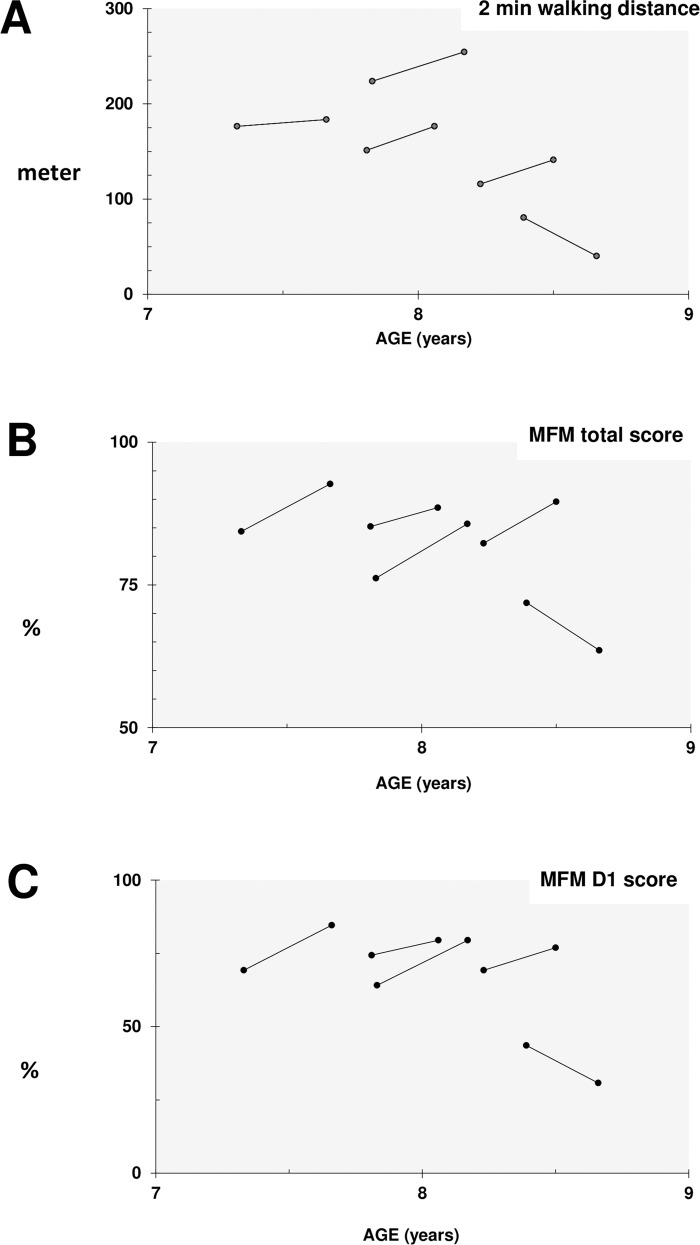
Clinical changes. Clinical changes over treatment period of 16 weeks are shown. Four of the five patients showed improvements in 2 min walking distance (A), the MFM total score (B) and the MFM D1 subscore (C).

In four of the five treated patients motor function and walking distances improved, while the oldest and most severely affected patient worsened after the treatment period. The mean total MFM and the MFM D1 subscore (standing and transfers) improved in the treated DMD patients over the period of 16 weeks by 3.6% (total MFM) and +6.2% (MFM D1). Also, the mean two minute walking distance improved (+9.6 meter) (Table 4). Individual changes are presented in Fig 4.

**Table 4:** Summary of clinical data.

	DMD Pretreatment		DMD Posttreatment		Change		
	Mean	(SD)	Mean	(SD)	Mean	(%)	*p* value*
**2 min walking distance, m**	149.6	55.11	159.2	78.1	9.6	6.4%	0.498
**Motor function measure**							
Total score, %	81.0	5.4	84.6	11.9	3.6%		0.414
D1 Subscore, %	64.1	12.0	70.3	22.3	6.2%		0.225
D2 Subscore, %	96.1	2.5	97.8	5.0	1.7%		0.450
D3 Subscore, %	86.7	5.2	88.6	6.4	1.9%		0.593

D1 Subscore = standing and transfers, D2 Subscore = axial and proximal motor capacity, D3 Subscore = distal motor capacity

*P values were calculated using the Wilcoxon signed-rank test

## Discussion

While patients with primary mitochondrial dysfunction disorders frequently display impaired muscle function [3], mitochondrial dysfunction is not generally considered to play a major role in the pathophysiology of DMD. However, our baseline data demonstrate a significantly reduced expression of mitochondrial proteins in DMD muscle samples, confirming the presence of severe mitochondrial abnormalities in DMD. Children with DMD have elevated synthesis of ADMA, an endogenous nNOS inhibitor, diminished arginine synthesis, and reduced NO bioavailability compared to healthy children [17]. Aim of this proof-of-concept investigator driven pilot-trial was to examine if increasing intramuscular NO concentrations would ameliorate DMD via stimulation of mitochondrial biogenesis. Therefore, we treated five ambulatory DMD patients for 16 weeks with L-arginine [34] a NO precursor, and metformin, a pharmacological AMPK activator and by this, indirect stimulator of nNOS [22, 35, 36]. Our treatment significantly increased nitrotyrosine concentrations and led to consistent cGMP increases in skeletal muscle biopsies of DMD patients indicating an increased NO formation in DMD muscle. In addition, a consistent increase of mitochondrial protein expression was observed. cGMP concentrations were positively correlated with mitochondrial protein concentrations in DMD muscle. Even more important, we also observed a positive correlation between the increase in NO concentration markers and mitochondrial protein expression, indicating a direct relation between intramuscular NO and subsequent mitochondrial protein expression in DMD muscle. It is often thought that NO, in general, might be harmful to skeletal muscle. NO generated by inducible nitric oxide synthase (iNOS) induces muscle atrophy via regulation of several transcription factors [37]. NO can react with superoxide anions (O^2-^) to form the toxic molecule peroxynitrite (ONOO-), which is believed to increase oxidative stress and muscle damage [38]. In contrast, neuronal and endothelial nitric oxide synthase (nNOS and eNOS, respectively) are expressed in the skeletal muscle and released NO in physiological amounts is known to protect cells from damage due to increased oxidative stress. There is no evidence that exclusively nNOS is stimulated in our study but the increase of NO markers but the parallel decrease of ROS markers in DMD muscle after treatment as well as the negative correlation of cGMP to carbonylated proteins indicates that L-arginine and metformin treatment decreases oxidative stress in muscle. If otherwise iNOS derived NO predominated to the observed CGMP and nitrotyrosine we would have expected an increase of oxidative stress and carbonylated proteins. We used metformin in its function as an AMPK activator and indirect nNOS stimulator, metformin has a wide range of other activities in energy and muscle metabolism that the sum of biochemical effects apart from nNOS stimulation can’t been completely foreseen at this point of time. Carbonylated proteins can be elevated in diabetes and metformin decreases carbonylated proteins in this condition indicating a reduction of oxidative stress. This can contribute to the anti-diabetic properties of the drug and also to the beneficial response observed in our study. Recently, elevated ADMA concentration were observed in metformin treated adult diabetic patients compared to untreated patients [39] Thus, one could suppose that metformin increases ADMA (nNOS inhibitor) leading to a reduced nNOS derived NO synthesis. However, metformin and ADMA are structural analogs that function as competitive antagonists on energy metabolism, protein synthesis, and growth [40]. Therefore, it seems more likely that ADMA may accumulate when ADMA nNOS interaction is blocked by metformin. We did not measure ADMA in this study but we did no observe any significant ADMA change in 6 patients with dystrophinopathy type Becker (the milder variant of dystrophinopathy) after 6 weeks of treatment with metformin (unpublished observation). Dystrophinopathy is a hypercatabolic condition with increased energy need early in the disease [41]. NO is capable of influencing oxidative energy metabolism via modulating the mitochondrial respiratory chain complex IV (cytochrome c oxidase). NO competes with molecular oxygen towards binding to the active site of cytochrome c oxidase, thereby inhibiting its activity and reducing oxygen consumption [42]. To assess the metabolic consequences of the treatment–in vivo—indirect calorimetry was performed. The change of used energy substrates from carbohydrates to fatty acids indicates altered metabolic and mitochondrial functions. The reduced resting energy expenditure per kg muscle / 24 h after treatment is consistent with an overall improved energetic situation. The latter is also mirrored by the improved clinical MFM scores and walking distances, while ambulatory DMD patients older than seven years usually show a high annual decrease of MFM total and D1 subscores [43]. Albeit the small number of patients, the mean changes of the MFM scores (MFM total score +3.6%, D1 subscore +6.2%) show an amelioration compared to published natural history data and data for the standard treatment of care with steroids. Untreated ambulatory DMD patients older than seven years showed a median annual total MFM decrease of -7.9% (D1 subscore -17.2%) as demonstrated by the group of C. Bérard in France 2010 [43]. The standard treatment of care with steroids lead to a mean improvement in that age group of only +1,9% of the MFM total score and +1.5% of the D1 subscore 3 months after therapy onset as investigated two years later in brazil [44]. Even if comparison might be affected by differences in standards of care in these countries the MFM is a validated clinical score that is reproducible, with good to excellent coefficients of the interrater reliability [27]. A key pathophysiological process in DMD consists in transformation of muscle into adipose tissue [12]. The increased fat utilization in DMD muscle after treatment could also result in slowed disease progression. An indication for a disease-slowing effect is provided by the thigh muscle MRI and whole body DEXA data which did not show any significant increase of the thigh muscle or body fat fractions. The increase in muscle fat fraction (MFF) of 1.5% in thigh muscles (1.3% in quadriceps) in 16 weeks, corresponding to a calculated annual increase of 4.5% (3.9% in quadriceps) contrasts with the 9.1% of annual muscle fat fraction (MFF) increase in thigh muscles (10.1% in quadriceps) in age-matched, untreated, ambulatory DMD patients[45]. While age-matched ambulant DMD patients typically show a reduction of -17.8% per year (quadriceps -19.5%), in our treated patients we surprisingly observed a trend towards an increase in healthy muscle area of 5.9% (quadriceps +4.7%); this should be further examined in a larger scale clinical trial.

In addition to the approach utilized in this trial, other possible NO-based strategies to treat DMD might exist. Since a major function of nNOS-derived NO is to stimulate cGMP production, it is conceivable that impaired nNOS function might be partially restored using phosphodiesterase 5 (PDE5) inhibitors such as sildenafil and tadalafil. In line with this model, PDE5 inhibition has been shown to increase cytosolic cGMP [46]. However, PDE5 inhibition using sildenafil could not show improved muscle function in Becker muscular dystrophy [47]. Another cGMP-independent, NO-dependent pathway involves peroxynitrite (ONOO-) formation, which is important for muscle protein synthesis and muscle hypertrophy [48]. Another potential strategy to stimulate NO-formation in DMD is the use of NO donors. In the mdx mouse model, a combination of ibuprofen and ISDN (isosorbide dinitrate, a NO donor) reduced muscle necrosis and inflammation and improved voluntary movements and resistance to exercise [49]. In contrast however, the combined approach of an NO-donating NSAID in human DMD patients resulted in no significant clinical improvement [50]. As our approach interferes more upstream in this pathway, it can also positively influence cGMP-independent NO-pathways. Finally, the advantage of L-arginine or L-citrulline is to show fewer side effects compared to PDE5 inhibitors that are typically associated with side effects such as headaches.

Our study has clear limitations due to the small number of patients, the short observation period, and the lack of placebo-treated controls. Unfortunately direct measurements of NO are not possible. As a consequence indirect NO markers have to be used. As nitrite / nitrate are only reliably measurable by mass spectroscopy we analysed cGMP (as second messenger of NO) and nitrotyrosine that increases when NO concentration increase. This approach has limitations as cGMP can also be influenced by other substances or phosphodiesterase inhibition. The measurement of nitrotyrosine has limitations, too. Nitrotyrosine levels can also be elevated during inflammation and increased oxidative stress when inducible NOS (iNOS) is activated leading to elevated NO concentrations. As L-arginine and metformin treatment decreased carbonylated proteins (marker of oxidative stress) the increase of cGMP and nitrotyrosine is unlikely to be linked to iNOS and inflammation, but this has not been proven. A further limitation of our study is that, despite the increase of cGMP, nitrotyrosine, and mitochondrial proteins after treatment we can’t exclude the possibility that the effects seen in our study may have also partially resulted from L-arginine effects beyond NO synthesis. L-arginine is involved in many physiological processes as recently investigated by Kayacelebi et al [51]. Thus other pathways could have been involved and could have contributed to the observed effects on muscle function. For example L-Arg is the substrate of AGAT which produces guanidine acetate, the substrate of GAMT and precursor of creatine, which can improve muscle energetics via increased phosphocreatine concentrations. One patient was treated with steroids. The treatment with metformin and L-arginine showed the same clinical and paraclinical results in this boy as in the other “responders”. However, a synergistic or additive effect to steroids is possible but cannot be substantiated or excluded at this time.

However, despite the restrictions, this proof-of-concept pilot study showed clear and consistent trends towards amelioration of muscular metabolism both *in vitro* and *in vivo*. In conclusion, our current data strongly suggest that targeting cellular NO levels using L-arginine and metformin is a promising new therapeutic strategy for the treatment of DMD. Evidently, further studies are needed to verify our results using larger patient cohorts and longer treatment periods. Therefore, we recently initiated a randomized placebo controlled double-blind study in DMD patients [52] to validate our therapeutic approach. Additionally we perform a pilot trial to extend this approach to ambulant patients with Becker muscular dystrophy [53].

## Supporting Information

S1 Appendix CONSORT checklist. (PDF)

S2 Appendix Study protocol in German. (PDF)

S3 Appendix Study protocol in English. (PDF)
