# Supplementary material for: Improved Muscle Function in Duchenne Muscular Dystrophy through L-Arginine and Metformin: An Investigator-Initiated, Open-Label, Single-Center, Proof-Of-Concept-Study
Source: PLoS One. 2016 Jan 22;11(1):e0147634. doi: 10.1371/journal.pone.0147634 (PMC4723144; doi:10.1371/journal.pone.0147634)
Supplement: S1 Appendix — (PDF) [file pone.0147634.s001.pdf]

# S1 CONSORT checklist of information to include when reporting a randomised trial

| Section/Topic | Item No | Checklist item | Reported on page No |
|---|---|---|---|
| **Title and abstract** | | | |
| | 1a | Information on how unit were allocated to interventions | **N.A.** |
| | 1b | Structured  abstract recommended | **2** |
| | 1c | Information on target population or study sample | **2** |
| **Introduction** | | | |
| Background | 2a | Scientific background and explanation of rationale | **3-4** |
| | 2b | Theories used in deigning behavioural interventions | **N.A.** |
| **Methods** | | | |
| Participants | 3a | Eligibility criteria for participants, including criteria at different levels in recruitment/sampling plan (e.g., cities, clinics, subjects) | **5** |
| | 3b | Method of recruitment (e.g., referral, self-selection), including the sampling method if a systematic sampling plan was implemented | **5** |
| | 3c | Recruitment setting | **5** |
| | 3d | Settings and locations where the data were collected | **5** |
| Interventions | 4a | Details of the interventions intended for each study condition and how and when they were actually administered, specifically including | **5-6** |
| | | - Content: what was given? | **5** |
| | | - Delivery method: how was the content given? | **5** |
| | | - Unit of delivery: how were the subjects grouped during delivery? | **N.A.** |
| | | - Deliverer: who delivered the intervention? | **6** |
| | | - Setting: where was the intervention delivered? | **6** |
| | | - Exposure quantity and duration: how many sessions or episodes or events were intended to be delivered? How long were they intended to last? | **5** |
| | | - Time span: how long was it intended to take to deliver the intervention to each unit? | **N.A.** |

| | | | |
|---|---|---|---|
| | | - Activieties to increase compliance or adherence (e.g., incentives) | **6** |
| Objectives | 5 | Specific objectives and hypotheses | **4** |
| Outcomes | 6a | Clearly defined primary and secondary outcome measures | |
| | 6b | Methods used to collect data and any methods used to enhance the quality of measurements | **6-9** |
| | | Information on validated instruments such as psychometric and biometric properties | **6-7** |
| Sample size | 7a | How sample size was determined and, when applicable, explanation of any interim analyses and stopping rules | **5** |
| Assignment Method | 8a | Unit of assignment | **N.A.** |
| | 8b | Methods used to assign units to study conditions, including details of any restriction | **N.A.** |
| | 8c | Inclusion of aspects employed to help minimize potential bias induced due to non-randomization | **N.A.** |
| Blinding | 9 | Whether or not participants, those administering the interventions, and those assessing the outcomes were blinded to study condition assignment; if so, statement regarding how the blinding was accomplished and how it was assessed. | **9-10 1** |
| Unit of Analysis | 10a | Description of the smallest unit that is being analysed to assess intervention effects | **5** |
| | 10b | If the unit of analysis differs from the unit of assignment, the analytical method used to account for this | **N.A.** |
| Statistical methods | 11a | Statistical methods used to compare study groups for primary methods outcome(s), including complex methods of correlated data | **9** |
| | 11b | Statistical methods used for additional analyses, such as a subgroup analysis and adjusted analysis. | **N.A.** |
| | 11c | Methods for imputing missing data, if used | **N.A.** |
| | 11d | Statistical software or programs used | **9** |

| | | | |
|---|---|---|---|
| **Results** | | | |
| Participant flow | 12a | Flow of participants through each stage of the study: enrolment, assignment, allocation, and intervention exposure, follow-up, analysis | **Fig. 1** |
| | 12b | Description of protocol deviations from study as planned, along with reasons | **Fig. 1** |
| Recruitment | 13 | Dates defining the periods of recruitment and follow-up | **6** |
| Baseline data | 14a | Baseline demographic and clinical characteristics of participants in each study condition | **N.A.** |
| Numbers analysed | 14b | Baseline characteristics for each study condition relevant to specific disease prevention research | **N.A.** |
| | 14c | Baseline comparison of those lost to follow-up and those retained, overall and by study condition | **N.A.** |
| | 14d | Comparison between study population at baseline and target population of interest | **9-20** |
| Baseline equivalence | 15 | Data on study group equivalence at baseline and statistical methods used to control for baseline differences | **9-20** |
| Numbers analyzed | 16a | Number of participants included in each analysis for each study condition, particularly when the denominators change for different outcomes; statement of the results in absolute numbers when feasible | **5** |
| | 16b | Indication of whether the analysis strategy was “intention to treat” or, if not, description of how non-compliers were treated in the analyses. | **N.A.** |
| Outcomes and estimation | 17a | For each primary and secondary outcome, a summary of results for each estimation study condition, and the estimated effect size and a confidence interval to indicate the precision | **N.A.** |
| | 17b | Inclusion of null and negative findings | **N.A.** |
| | 17c | Inclusion of results from treating pre-specified causal pathways through which the intervention was intended to operate, if any | **9-20** |

| | | | |
|---|---|---|---|
| Ancillary analyses | 18 | Summary of other analyses performed, including subgroup or restricted analyses, indicating which are pre-specified or exploratory | **N.A.** |
| Adverse events | 19 | Summary of all important adverse events or unintended effects in each study condition | **6** |
| **Discussion** | | | |
| Interpretation | 20a | Interpretation of the results, taking into account study hypotheses, sources of potential bias, imprecision of measures, multiplicative analyses, and other limitations or weaknesses of the study | **21-24** |
| | 20b | Discussion of results taking into account the mechanism by which the intervention was intended to work (causal pathways) or alternative mechanisms or explanations | **21-24** |
| | 20c | Discussion of the success of and barriers to implementing the intervention, fidelity of implementation | **21-24** |
| | 20d | Discussion of research, programmatic, or policy implications | **24** |
| Generalizability | 21 | Generalizability (external validity) of the trial findings, taking into account the study population, the characteristics of the intervention, length of follow-up, incentives, compliance rates, specific sites /settings involved in the study, and other contextual issues | **24** |
