# Supplementary material for: Improved Muscle Function in Duchenne Muscular Dystrophy through L-Arginine and Metformin: An Investigator-Initiated, Open-Label, Single-Center, Proof-Of-Concept-Study
Source: PLoS One. 2016 Jan 22;11(1):e0147634. doi: 10.1371/journal.pone.0147634 (PMC4723144; doi:10.1371/journal.pone.0147634)
Supplement: S2 Appendix — (PDF) [file pone.0147634.s002.pdf]

STUDIENPROTOKOLL

1. Titel des Studienprotokolls

Pilotstudie zur Untersuchung der Wirksamkeit von L-Arginin und Metformin bei Kindern zwischen 7 und 10 Jahren mit Muskeldystrophie Duchenne

Version 2, 17. Oktober 2011

Prüfer:

Name/Titel:	PD Dr. med. Dirk Fischer / Oberarzt
Abteilung	Neuropädiatrie
Spital	Universitätskinderklinik beider Basel

Unterschrift: .... Datum: 19.04.2012

Co-Prüfer:

Dr. med. Patricia Hafner, Assistenzärztin Neurologie und Neuropädiatrie, Basel

Liste der Mitarbeiter

Prof. Dr. med. Peter Weber, Leiter der Neuropädiatrie, Universitätskinderklinik beider Basel

Dr. med. Urs Pohlman, Assistenzarzt Neuropädiatrie, Basel

Dr. med Arne Fischmann, Oberarzt Neuroradiologie, Basel

2. Hintergrundinformation Arzneimittel

2.1 Bezeichnung und Indikation der Prüfpräparate

L-Arginin

L-Arginin gilt als eine halb-essentielle Aminosäure, die durch den menschlichen Organismus selbst synthetisiert werden kann. Allerdings sind die entstehenden Mengen nicht ausreichend, um den Bedarf vor allem bei heranwachsenden Menschen vollständig zu decken. Daher ist L-Arginin für Kinder essentiell, muss also durch die Nahrung aufgenommen werden. Vor allem in Nüssen und Kernen, wie z.B. Walnüssen oder Kürbiskernen, aber auch in tierischen Produkten wie Schweinefleisch oder Lachs ist Arginin in relevanten Konzentrationen enthalten. Bei einer Eiweisszufuhr von etwa 70–90 g/Tag ergibt sich eine rechnerische tägliche Argininzufuhr von ca. 2–5 g/Tag.

L-Arginin ist wichtiger Metabolit des Harnstoffzyklus. Als alleinige Vorstufe wird es hier durch Stickstoffmonoxid-Synthase (NOS) in Stickstoffmonoxid (NO) umgewandelt. Eine Erhöhung von L-Arginin im Plasma führt zu vermehrter Bildung von NO, das Bildung und Funktion der Mitochondrien im Skelettmuskel stimuliert (1).

L-Arginin ist kein Arzneimittel, sondern ein Nahrungsmittelzusatz und untersteht deshalb nicht dem Arzneimittelgesetz.

Metformin (Metfin®)

Metformin (Metfin®) ist ein orales Biguanid-Antidiabetikum. Es ist von Swissmedic zugelassen für die Behandlung einer erhöhten Insulinresistenz und Therapie des Typ 2-Diabetes, insbesondere bei übergewichtigen Patienten, deren erhöhter Blutzucker sich durch eine Diät und körperliche Aktivität alleine nicht kontrollieren lässt. Die blutzuckersenkende Wirkung von Metformin beruht primär auf einem die Insulinresistenz durchbrechenden Effekt in Leber und Muskel. Es senkt nur in Gegenwart von Insulin sowohl den basalen als auch den postprandialen Plasma-Glukosespiegel. Metformin hat keine stimulierende Wirkung auf die Insulinsekretion und löst bei alleiniger Anwendung keine Hypoglykämie aus (2).

In der EU und in der Schweiz ist Metformin seit 2004 für diese Indikationen auch bei Kindern ab 10 Jahren zugelassen. Die Sicherheit und Wirksamkeit der Anwendung von Metformin bei Kindern und Jugendlichen wurden vor der Zulassung in einer randomisierten Doppelblind-Studie an 82 Kindern und Jugendlichen im Alter von 10–16 Jahren geprüft. Neueste Daten an 53 Insulin-resistenten Kindern im Alter von 6-12 Jahren (davon 19 im Alter von 6-10 Jahren) wiesen eine gute Verträglichkeit auf (3). Schwerwiegende Nebenwirkungen traten in der angewandten Dosierung von 2000 mg Metformin täglich nicht auf.

2.2 Dosierung, Dosierungsschema, Behandlungsdauer

L-Arginin

Die Normwerte der Plasmakonzentration von L-Arginin liegen bei Kindern von 8-11 Jahren bei 54.29 bis 115.33 µmol/l. Ziel ist es mittels Substitution eine Verdopplung der Konzentration (minimal 100 µmol/l- maximal 200 µmol/l) zu erreichen. Durch die orale Gabe von L-Arginin 3 x 2,5g bzw. 3 x 5g/m^{2}KOF/d konnten Bennett et al. (19) bei Kindern im Alter von 7-17 Jahren eine Verdopplung der Plasmakonzentration von L-Arginin erreichen. Dies entspricht ebenfalls der Dosis von 3 x 0,3-0,5mg/kgKG/d, die Koga et al. (20). Zur Erhöhung der Plasmakonzentration von L-Arginin >100 µmol/l bei Patienten mit MELAS eingesetzt haben.

In dieser Studie wird daher eine Dosis von 3 x 2.5g pro Tag während der Dauer von 16 Wochen verabreicht.

Metformin (Metfin®)

Unpublizierte eigene Erfahrungen zeigen, dass bei Knaben mit Muskeldystrophie Duchenne oft eine Insulinresistenz nachweisbar ist, womit formal auch eine Behandlung der zugelassenen Indikation möglich wäre. Für die geplante Studie ist eine Dosierung von 2 Filmtabletten à 250 mg zweimal täglich während einer Dauer von 16 Wochen vorgesehen. Diese Dosis verspricht ein möglichst geringes Auftreten von gastrointestinalen Nebenwirkungen bei genügender Aktivierung des Muskelmetabolismus. Unter Einnahme von Metformin kann es zu Beginn der Behandlung zu Übelkeit, Erbrechen, Durchfall und Bauchschmerzen kommen. Diese Symptome gehen meist spontan zurück.

3. Zielsetzungen und Zweck

3.1 Hintergrund, Begründung und Ziel der Studie

Die Muskeldystrophie vom Typ Duchenne (DMD) ist eine X-chromosomal erbliche Erkrankung, die etwa bei einem von 3500 Jungen auftritt. Ursache der DMD sind Veränderungen im Dystrophin-Gen, die zu einem Fehlen des Dystrophin Proteins führen. Dystrophin liegt an der Innenfläche der muskulären Plasmalemm und interagiert als Strukturprotein sowohl mit Membranproteinen, welche den Dystrophin-assoziierten Glykoprotein-Komplex (DGC) bilden, als auch mit Proteinen des Zellskeletts. Bei Fehlen von Dystrophin kommt es zu einer Membraninstabilität der Muskelzelle, die zu einem verfrühten Untergang der Muskelfasern führt. Die zerstörten Muskelfasern werden zunehmend durch Binde- und vor allem durch Fettgewebe ersetzt. Klinisch kommt es zunächst im Alter von 3-4 Jahren zu einer verzögerten motorischen Entwicklung. Im Verlauf der Erkrankung nimmt die Muskelschwäche zu, so dass die Patienten um das zehnte Lebensjahr rollstuhlpflichtig

werden. Sie entwickeln eine Skoliose, in einem späteren Stadium eine Ateminsuffizienz und eine Kardiomyopathie. Die Lebenserwartung hat sich in den letzten Jahren beträchtlich verbessert, unter anderem dank der nicht-invasiven Ventilation.

Bei Verlust von Dystrophin bei der DMD kommt es zu einem Störung des DGC und Zerstörung der gesamten Zellmembranstruktur. Dieser strukturelle Defekt führt zu einem vermehrten Einstrom von Kalziumionen, Aktivierung von Proteasen wie z.B. Calpainen (Ca-abhängigen Proteasen) und einer erhöhten Produktion von reaktiven Sauerstoffspezies (ROS) führt, welche wiederum die Zerstörung der Zellmembran fördern. ROS, welche bei DMD Patienten stark erhöht sind, entstammen vorwiegend aus Mitochondrien, was für eine gestörte Mitochondrienfunktion bei den betroffenen Patienten spricht. Sperl et al. berichteten von einer verminderten Oxidationsrate in Muskelbiopsien von DMD Patienten (5). Die mitochondriale Dysfunktion wurde ebenfalls in der *mdx*-Maus untersucht. Untersuchungen am *mdx* Skelettmuskel ergaben, verglichen mit gesunden Kontrollen, eine 50%ige Reduktion der am Atemzyklus beteiligten Enzyme (6). Die Autoren stellten ebenfalls fest, dass von mdx-Muskel isolierte Mitochondrien lediglich 60% der normalen Respirationsrate erreichen. Zuletzt demonstrierten Millay et al. einen starken Zusammenhang zwischen Mitochondrien-abhängiger Nekrose und Muskeldystrophie in mehreren Mausmodellen (inkl. dem mdx-Modell), was nahe legt, dass Mitochondrien eine tragende Rolle spielen in der Pathogenese der Muskeldystrophie vom Typ Duchenne (7).

Der Verlust von Dystrophin führt ebenfalls zu erheblichen metabolischen Veränderungen. Bei Patienten mit Muskeldystrophie Duchenne konnte eine fast 80%ige Reduktion der Dystrophin-assoziierten neuronalen Stickstoffmonoxidsynthase (nNOS) beobachtet werden, was wiederum zu einer verminderten Produktion von intramuskulärem Stickstoffmonoxid (NO) führt (8). Zusätzlich ist Arginase II, welche mit nNOS um L-Arginine konkurriert, bei DMD-Patienten stark erhöht (9) was wiederum die Menge des intrazellulär verfügbaren Stickstoffmonoxids (NO) reduziert (Fig 1a, b). In gesunden Kontrollpersonen wird die intramuskuläre NO Produktion bei körperlicher Belastung durch eine verstärkte nNOS-Aktivität stark erhöht. NO erhöht die Glucose-Aufnahme in die Muskelzelle, stimuliert die Mitochondrienfunktion und Biogenese in der Skelettmuskulatur (10).

Daher scheinen erhöhte NO Konzentrationen vielversprechend zu sein, um die verheerende Auswirkung des fehlenden Dystrophins bei Patienten mit Muskeldystrophie Duchenne durch Stimulation der muskulären Energieproduktion sowie der mitochondrialen Funktion zu verbessern. Direkte NO-Spender verbessern den Effekt von Prednison (dem einzigen etablierten Medikament, das eine Verzögerung der Muskeldystrophie bewirkt) im Mausmodell an *mdx*-Mäusen (13). Zudem kann die Expression eines Muskel-spezifischen nNOS Transgens die Ausdauer von mdx-Mäusen während des Laufens auf dem Laufrad erhöhen (15). Aber auch eine Behandlung mit L-Arginin führte über eine indirekte Erhöhung von NO zu einer verbesserten muskulären Funktion bei *mdx*-Mäusen (14).

AMPK ist ein weiterer wichtiger Regulator der muskulären Energiebalance. AMPK hat sich als Schlüsselenzym der oxidativen Funktion der Skelettmuskulatur, inklusive der metabolischen Enzymexpression und mitochondrialen Biogenese, herausgestellt (12). Es agiert teilweise durch Phosphorylierung und Aktivierung von nNOS (11). nNOS kann durch AMPK aktiviert werden, welches wiederum pharmakologisch durch Metformin stimuliert wird (16)(Fig. 1c).

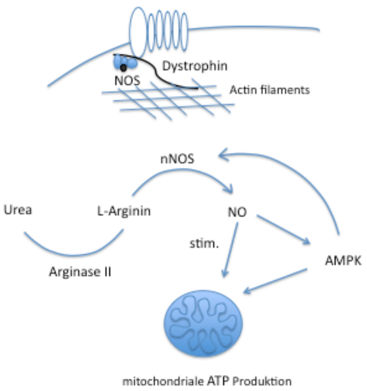

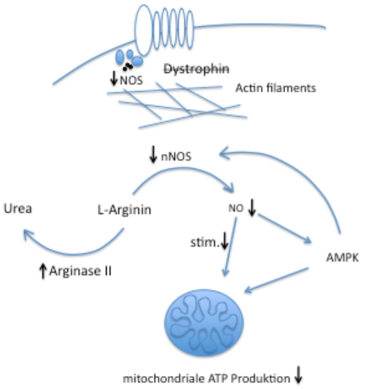

a) normale Muskelzelle

b) Muskeldystrophie Duchenne, unbehandelt

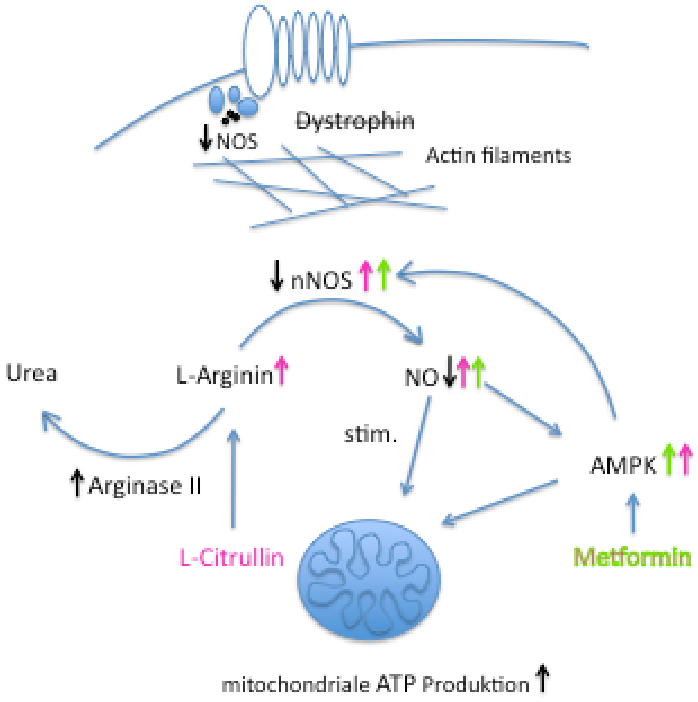

c) Muskeldystrophie Duchenne, behandelt mit L-Arginin und Metformin

3.2 Fragestellung, Studienpopulation

In dieser Studie ist wird untersucht, ob eine kombinierte Behandlung mit L-Arginin und Metformin, welches nNOS stimuliert, zu einer Erhöhung des Energiemetabolismus im Muskel von Patienten mit Muskeldystrophie vom Typ Duchenne führt und somit eine Verbesserung der Muskelkraft und Verminderung einer vorzeitigen Erschöpfbarkeit bewirkt und zu einer Verlangsamung des Muskelabbaus bei Patienten mit Muskeldystrophie Duchenne führt. Langfristig soll so eine Verlängerung der Zeit bis zum Verlust der Gehfähigkeit erreicht werden.

Zunächst möchten wir die Wirksamkeit von L-Arginin und Metformin im Rahmen einer kleinen Pilotstudie an ca. 5-6 DMD Patienten im Alter zwischen 7 und 10 Jahren, also bei Patienten mit noch erhaltener Gehfähigkeit, mittels klinischen, bildgebenden sowie immunhistochemischen Untersuchungen nachweisen.

3.3 Hypothese

Zusammengefasst sollten beide Ursachen, der erhöhte L-Arginin Abbau durch Arginase II und die verminderte NO Produktion durch reduziertes nNOS behandelt werden, um die NO-Konzentration zu erhöhen, um somit auch eine Verbesserung der Muskelkraft, eine Verlangsamung des Muskelabbaus und langfristig eine Verlängerung der Zeit bis zum Verlust der Gehfähigkeit zu erreichen.

4. Studiendesign

4.1 Hauptzielparameter und sekundäre Zielparameter

Hauptzielparameter

In vivo – Messung des Muskelmetabolismus

Der Anteil des gesamten Fett- und Muskelgehaltes (lean body mass) des Körpers wird mittels Dexametrie (dual-energy X-ray absorptiometry) gemessen. Dies ist eine nicht-invasive Methode mittels derer der Patient mit schwach dosierten Röntgenstrahlen durchleuchtet wird. Fett, Muskeln und Wasser schwächen die Strahlen unterschiedlich ab. Aus der Abschwächung berechnet der Computer präzise die Körperzusammensetzung.

Der muskuläre Energiemetabolismus (metabolischer Grundumsatz, Oxigenierung von Fett und Kohlenhydraten beim nüchternen Patienten) erfolgt mittels Kalorimetrie.

Sekundäre Zielparameter

1. In vivo - Messung des Fettgehalts der Muskulatur mittels MRI

Muskel-MRI ist eine vielversprechende Methode, um die Wirksamkeit von therapeutischen Interventionen zur Verlangsamung des fettigen Umbaus der Muskulatur zu messen. Die Untersuchung mittels MRI hat mehrere Vorteile: sie ist nicht invasiv, schnell und kann die relevanten muskulären Strukturen ideal abbilden. Mit speziell entwickelten Sequenzen (Dixon-Methode) kann der fettige Umbau der Muskulatur gemessen und im Verlauf beurteilt werden. Die Untersuchungszeit im MRI wird insgesamt ca. 20 Minuten betragen. Es erfolgt keine Gabe von Kontrastmittel. Die Untersuchung erfolgt ohne Narkose.

Die Durchführung erfolgt in Zusammenarbeit mit dem Departement für Radiologie, Universitätsspital Basel und wird ebenfalls zu Studienbeginn sowie nach 16-wöchiger Behandlungsphase durchgeführt.

2. In vitro – Messung des Muskelmetabolismus

Zu Beginn der Studie sowie nach 16-wöchiger medikamentöser Behandlung erfolgt eine Muskelbiopsie aus dem Musculus vastus lateralis am Oberschenkel. Die Biopsie wird in Anästhesie durch einen Kinderchirurgen entnommen. Die histopathologische Untersuchung umfasst NO-Analysen und wird im Departement für Neuropathologie der Universität Basel durchgeführt (PD Dr. Stephan Frank). Analysen der Konzentration des aktivierten, phosphorylieren pAMPK im entnommenen Muskel erfolgen durch Prof. Ch. Handschin, Biozentrum Basel.

3. Klinische Messung der Muskelkraft

Der MFM (motor function measurement) ist ein validierter Test zur Messung der Muskelkraft in gehfähigen und rollstuhlgebundenen DMD Patienten. Dieser Test dient als Verlaufsparameter, um das Fortschreiten der Muskelschwäche zu erfassen. Zusätzlich zu diesem standardisierten Verfahren wird die Kraft für den Faustschluss, Ellbogenstreckung und Ellbogenbeugung, Hüftstreckung und Hüftbeugung sowie Fusshebung mittels Myometrie im Rahmen einer physiotherapeutischen Untersuchung erfasst.

4. Laborchemische Parameter

Differentialblutbild

Chemie:	Transaminasen, Kreatinin, Elektrolyte, Harnstoff
Marker der Muskelnekrosen:	Kreatinkinase
Aminosäuren:	L-Citrullin und L-Arginin
Glucosestoffwechselmarker:	Nüchtern-Glucose, Nüchtern-Insulin (Insulin-Resistenz), Hba1c
Fettstoffwechselmarker:	HDL, LDL, Triglyceride, Adiponectin, Leptin
Ausschluss Lactatazidose:	Blutgasanalyse, Laktat-Spiegel, Laktat-Pyruvat-Quotient

4.2 Studiendesign

Bei dieser Studie handelt es sich eine Pilotstudie. Der Flowchart der Studie ist unten abgebildet. 5-6 Knaben im Alter von 7-10 Jahren sollen im Zeitraum von Oktober 2011 bis Januar 2012 in die Studie eingeschlossen und während 16 Wochen behandelt werden.

**Screening**	**Visite 1 / Tag 1**	**Visite 2 / Woche 2**	**Visite 3 / Woche 4**	**Visite 4 / Woche 8**	**Visite 5 / Woche 16**
Patienteninformation	Körperliche Untersuchung	Körperliche Untersuchung	Körperliche Untersuchung	Körperliche Untersuchung	Körperliche Untersuchung
Einverständniserklärung	Vitalzeichen	Vitalzeichen	Vitalzeichen	Vitalzeichen	Vitalzeichen
Ein- und Ausschluss-kriterien	Blutentnahme	Blutentnahme	Blutentnahme	Blutentnahme	Blutentnahme
	Dexametrie / Kalorimetrie				Dexametrie / Kalorimetrie
	Muskelkraft				Muskelkraft
	MRI				MRI
	Muskelbiopsie				Muskelbiopsie
	Abgabe Studienmedika-mente				
	Abgabe Tagebuch	Kontrolle Tagebuch	Kontrolle Tagebuch	Kontrolle Tagebuch	Kontrolle und Rücknahme Tagebuch

Screening

Die Ein- und Ausschlusskriterien werden geprüft. Sind die Kriterien erfüllt und besteht der Wunsch zur Studienteilnahme, wird nach Unterschrift der Einverständniserklärung der Patient in die Studie eingeschlossen und für einen eintägigen Aufenthalt im Universitätskinderspital beider Basel aufgeboten.

Visite 1: Eintägige Hospitalisation

Es erfolgt eine Anamnese und die körperliche Untersuchung durch den Prüfarzt. Die Studienschwester misst danach den Blutdruck und den Puls. Dann wird eine Blutentnahme gemacht. Die Blutprobe wird ca. 15 ml enthalten, das entspricht in etwa 3 Esslöffeln. Es wird ein Marker des Muskelzelltodes bestimmt, die Kreatinkinase (CK), die Aminosäuren L-Citrullin und L-Arginin, die Glucosestoffwechselmarker (Nüchtern-Glucose, Nüchtern-Insulin, Hba1c), die Fettstoffwechselmarker (HDL, LDL, Triglyceride, Adiponectin, Leptin), zum Ausschluss einer Lactatazidose (eine arterielle Blutgasanalyse, Laktat, Laktat-Pyruvat-Quotient), die Blutchemie mit Leber- und Nierenwerten (GOT GPT, Kreatinin, Harnstoff) und den Elektrolyten sowie das Differentialblutbild, bestehend aus Blutplättchen, den roten und weissen Blutkörperchen.

Danach wird eine Dexametrie vorgenommen, um den Anteil des gesamten Fett- und Muskelgehaltes des Körpers zu messen. Bei dieser Methode wird der Patient mit schwach dosierten Röntgenstrahlen durchleuchtet. Fett, Muskeln und Wasser schwächen die niedrig

dosierten Röntgenstrahlen unterschiedlich ab. Aus der Abschwächung berechnet der Computer die Körperzusammensetzung ganz genau. Anschliessend erfolgt die Kalorimetrie zur Messung des muskulären Energiehaushalts.
Eine Physiotherapeutin wird die Muskelkraft des Patienten messen. Dies wird mittels eines international validierten Tests (motor function measurement) und mittels klinischer Tests, bei denen die notwendige Zeit bzw. Strecke für bestimmte Fähigkeiten gemessen werden (Aufstehen aus liegender Position, 10 m Gehstrecke und falls möglich die 6 Minuten Gehstrecke).
Danach folgt eine Magnetresonanztomographie der Beine. Die MRI Untersuchung bestimmt den Fettgehalt der Ober- und Unterschenkelmuskulatur rasch und zuverlässig. Die Messzeit für die Ober- und Unterschenkelmuskulatur beträgt jeweils rund drei Minuten. In diesen zweimal drei Minuten müssen die Kinder die Beine ruhig halten, da sonst die Untersuchungen nicht auswertbar sind. Auf eine Sedierung oder Narkose wird aufgrund der kurzen Untersuchungszeit grundsätzlich verzichtet. Die Gesamtuntersuchungszeit im MRI, inklusive Vorbereitung und Lagerung, beträgt somit wahrscheinlich rund 20 min. Sie dürfen selbstverständlich während der gesamten Zeit Ihr Kind begleiten und anwesend sein.
Als letzte Untersuchung wird eine Muskelbiospie durchgeführt. Dafür muss ein kleines Muskelstück aus dem äusseren Oberschenkelmuskel entnommen werden. Die Biopsie wird in Anästhesie durch einen Kinderchirurgen entnommen und dauert etwa 20 Minuten. Dieses kleine Muskelstück wird dann unter dem Mikroskop untersucht und dient dazu, die minimalen Veränderungen in den Muskeln zu sehen.
An diesem Tag werden ebenfalls die Medikamente für die gesamte Studiendauer verteilt.
Am nächsten Tag darf der Patient das Spital wieder verlassen.

Visite 2: Woche 2 (+/- 3 Tage, ambulant oder telefonisch, falls der Patient mehr als 500km von Basel entfernt wohnt)
Es erfolgt eine Anamnese und die körperliche Untersuchung durch den Prüfarzt oder den beauftragten Kinderarzt in der Nähe des Wohnortes des Patienten, der an der Studie teilnimmt. Die Studienschwester oder der Kinderarzt misst danach den Blutdruck und den Puls. Danach wird eine Blutentnahme gemacht. Die Blutprobe wird ca. 15 ml enthalten, das entspricht in etwa 3 Esslöffeln. Es wird ein Marker des Muskelzelltodes bestimmt, die Kreatinkinase (CK), die Aminosäuren L-Citrullin und L-Arginin, die Glucosestoffwechselmarker (Nüchtern-Glucose, Nüchtern-Insulin, Hba1c), die Fettstoffwechselmarker (HDL, LDL, Triglyceride, Adiponectin, Leptin), zum Ausschluss einer Lactatazidose (eine arterielle Blutgasanalyse, Laktat, Laktat-Pyruvat-Quotient), die Blutchemie mit Leber- und Nierenwerten (GOT GPT, Kreatinin, Harnstoff) und den Elektrolyten und das Differentialblutbild, bestehend aus Blutplättchen, den roten und weissen Blutkörperchen. Falls die Blutentnahme beim Kinderarzt in der Nähe des Wohnortes des

Patienten gemacht wurde wird das Blut zur Untersuchung in Studienzentrum nach Basel geschickt.

Visite 3: Woche 4 (+/- 3 Tage, ambulant oder telefonisch falls der Patient mehr als 500km von Basel entfernt wohnt)
Es erfolgt eine Anamnese und die körperliche Untersuchung durch den Prüfarzt oder den beauftragten Kinderarzt in der Nähe des Wohnortes des Patienten, der an der Studie teilnimmt. Die Studienschwester oder der Kinderarzt misst danach den Blutdruck und den Puls. Danach wird eine Blutentnahme gemacht. Die Blutprobe wird ca. 15 ml enthalten, das entspricht in etwa 3 Esslöffeln. Es wird ein Marker des Muskelzelltodes bestimmt, die Kreatinkinase (CK), die Aminosäuren L-Citrullin und L-Arginin, die Glucosestoffwechselmarker (Nüchtern-Glucose, Nüchtern-Insulin, Hba1c), die Fettstoffwechselmarker (HDL, LDL, Triglyceride, Adiponectin, Leptin), zum Ausschluss einer Lactatazidose (eine arterielle Blutgasanalyse, Laktat, Laktat-Pyruvat-Quotient), die Blutchemie mit Leber- und Nierenwerten (GOT GPT, Kreatinin, Harnstoff) und den Elektrolyten und das Differentialblutbild, bestehend aus Blutplättchen, den roten und weissen Blutkörperchen. Falls die Blutentnahme beim Kinderarzt in der Nähe des Wohnortes des Patienten gemacht wurde wird das Blut zur Untersuchung in Studienzentrum nach Basel geschickt.

Visite 4: Woche 8 (+/- 3 Tage, ambulant oder telefonisch falls der Patient mehr als 500km von Basel entfernt wohnt)
Es erfolgt eine Anamnese und die körperliche Untersuchung durch den Prüfarzt oder den beauftragten Kinderarzt in der Nähe des Wohnortes des Patienten, der an der Studie teilnimmt. Die Studienschwester oder der Kinderarzt misst danach den Blutdruck und den Puls. Danach wird eine Blutentnahme gemacht. Die Blutprobe wird ca. 15 ml enthalten, das entspricht in etwa 3 Esslöffeln. Es wird ein Marker des Muskelzelltodes bestimmt, die Kreatinkinase (CK), die Aminosäuren L-Citrullin und L-Arginin, die Glucosestoffwechselmarker (Nüchtern-Glucose, Nüchtern-Insulin, Hba1c), die Fettstoffwechselmarker (HDL, LDL, Triglyceride, Adiponectin, Leptin), zum Ausschluss einer Lactatazidose (eine arterielle Blutgasanalyse, Laktat, Laktat-Pyruvat-Quotient), die Blutchemie mit Leber- und Nierenwerten (GOT GPT, Kreatinin, Harnstoff) und den Elektrolyten und das Differentialblutbild, bestehend aus Blutplättchen, den roten und weissen Blutkörperchen. Falls die Blutentnahme beim Kinderarzt in der Nähe des Wohnortes des Patienten gemacht wurde wird das Blut zur Untersuchung in Studienzentrum nach Basel geschickt.

Visite 5: Woche 16 (+/- 3 Tage, eintägige Hospitalisation)
Es erfolgt eine Anamnese und die körperliche Untersuchung durch den Prüfarzt Die Studienschwester misst danach den Blutdruck und den Puls. Danach wird eine Blutentnahme gemacht. Die Blutprobe wird ca. 15 ml enthalten, das entspricht in etwa 3 Esslöffeln. Es wird ein Marker des Muskelzelltodes bestimmt, die Kreatinkinase (CK), die Aminosäuren L-Citrullin und L-Arginin, die Glucosestoffwechselmarker (Nüchtern-Glucose, Nüchtern-Insulin, Hba1c), die Fettstoffwechselmarker (HDL, LDL, Triglyceride, Adiponectin, Leptin), zum Ausschluss einer Lactatazidose (eine arterielle Blutgasanalyse, Laktat, Laktat-Pyruvat-Quotient), die Blutchemie mit Leber- und Nierenwerten (GOT GPT, Kreatinin, Harnstoff) und den Elektrolyten und das Differentialblutbild, bestehend aus Blutplättchen, den roten und weissen Blutkörperchen.
Danach wird eine Dexametrie vorgenommen, um den Anteil des gesamten Fett- und Muskelgehaltes des Körpers zu messen. Anschliessend erfolgt die Kalorimetrie zur Messung des muskulären Energiehaushalts.
Eine Physiotherapeutin wird die Muskelkraft des Patienten messen (wie bei Visite 1 beschrieben).
Danach folgt eine Magnetresonanztomographie der Beine (wie bei Visite 1 beschrieben).
Als letzte Untersuchung wird eine Muskelbiospie durchgeführt (wie bei Visite 1 beschrieben).
Die Studie ist nun für den Patienten abgeschlossen.

Patienten (oder ihre Eltern) können von der Studie jederzeit freiwillig zurücktreten. Hierbei sollte allerdings der Prüfer versuchen, den Hauptgrund festzustellen und diese Information im CRF notieren.

4.2.1 Abbruchkriterien

Zusälich muss die Studie eingestellt werden, wenn entsprechende Ereignisse auftreten.
Folgenden Bedingungen/ Ereignisse können zu einem Studienabbruch führen:
- serious adverse event
- Zurücknahme der Zustimmung zur Studienteilnahme
- Gebrauch nicht erlaubter Medikation (siehe o.g. Ausschlusskriterien)
- Protokollabweichung von Seiten der Patienten (Malcompliance)
- logistische Gründe (Ortwechsel, des Patienten etc.)
- Umständee, die einen regelmäigen Besuchen der Kontrolltermine nicht mehr ermöglichen
- anormale Laborwerte einschliesslich Leber- oder Nierenfunktionstests (Erhöhung der Transaminasen > 200% der Norm, Kreatinin auf > 200% der Norm)
- Blutdruckanstieg > 95 Perzentile der Altersnorm
- Auftreten einer Hyperlactatazidose (> 4 mmol/l, pH < 7,25)

4.3 Massnahmen zur Bias Minimierung

Die Patienten werden nach dem DMD Register der Schweiz in alphabetischer, systematischer Reihenfolge nach Einschluss- und Ausschlusskriterien gesucht und für eine Studienteilnahme angefragt. Randomisierung und Verblindung erfolgen keine.

Die Visiten inklusive Anamnese und körperliche Untersuchungen erfolgen jeweils durch nur einen Prüfarzt. Die Physiotherapeutinnen, welche die Untersuchungen durchführen, wurden in Lyon, wo MFM etabliert und validiert wurde, ausgebildete und zertifiziert.

5. Auswahl der Versuchspersonen

5.1 Rekrutierung

Die Studienpatienten werden unter den betroffenen Knaben aus der neurologischen Sprechstunde des UKBB's sowie dem schweizerischen und dem DMD – Register rekrutiert. Derzeit sind im Schweizer Register ca. 120 DMD Patienten registriert.

5.2 Einschlusskriterien

- Patienten mit molekulargenetisch gesicherter Diagnose einer DMD
- Alter von 7-10 Jahren zum Zeitpunkt des Studieneinschlusses
- erhaltene Gehfähigkeit zum Zeitpunkt des Studieneinschlusses

5.3 Ausschlusskriterien

- Teilnahme an einer therapeutischen Studie für DMD innerhalb der letzten 3 Monate
- Einnahme von L-Arginin, L-Citrullin oder Metformin innerhalb der letzten 3 Monate
- Aktuelle Behandlung mit Steroiden (Deflazacort, Prednison)
- Andere chronische Erkrankung oder signifikante Einschränkung der Nieren-, Leber-, Herz-, Lungenfunktion nach Ermessen des Prüfarztes
- Bekannte Überempfindlichkeit auf L-Arginin oder Metformin

6. Bewertung der Wirksamkeit

1. Messung des Muskelmetabolismus mittels Dexametrie und Kaloimetrie am Tag 1 und zum Zeitpunkt von Woche 16.
2. Messung des Fettgehalts der Muskulatur mittels MRI am Tag 1 und zum Zeitpunkt von Woche 16.
3. Messung des Muskelmetabolismus mittels histologischer Untersuchung eines Muskelpräparates am Tag 1 und zum Zeitpunkt von Woche 16.
4. Klinische Messung der Muskelkraft mittels physiotherapeutischem Assessment am Tag 1 und zum Zeitpunkt von Woche 16.

7. Bewertung der Sicherheit

7.1 Sicherheitsparameter: Messmethoden und Zeitpunkte

Anlässlich jeder Visite ist eine klinische Untersuchung und eine Messung der Vitalparameter Blutdruck und Puls vorgesehen. Am Zeitpunkt Tag 1 und zum Zeitpunkt von Woche 16 sind laborchemische Kontrollen der folgenden Parameter vorgesehen:

Differentialblutbild, da unter der Einnahme von Metformin im Rahmen der Zulassungsstudien vereinzelte Fälle von Leukopenie, Thrombopenie und hämolytische Anämie gefunden wurden, Chemie (Transaminasen, Kreatinin, Elektrolyte, Harnstoff), Marker der Muskelnekrosen (Kreatinkinase), Aminosäuren (L-Arginin), Glucosestoffwechselmarker (Nüchtern-Glucose, Nüchtern-Insulin, Hba1c), Fettstoffwechselmarker (HDL, LDL, Triglyceride, Adiponectin, Leptin). Zum Ausschluss einer

Lactatazidose, welche durch die Einnahme von Metformin vorkommen könnte, werden eine arterielle Blutgasanalyse, Laktat-Spiegel und Laktat-Pyruvat-Quotient bestimmt.

Bei klinisch signifikanten Veränderungen (Anstieg von Kreatinin und der Transaminasen >200% des Normwertes, Laktatkonzentration von >8mmol/l) muss Metformin abgesetzt werden.

Sollten sich pathologische Zufallsveränderungen unabhängig von der bekannten Muskelerkrankung nachweisen lassen, werden die betroffenen Patienten selbstverständlich unverzüglich darüber informiert und entsprechend des aktuellen medizinischen Wissens über die Möglichkeiten zur weiteren Abklärung bzw. Behandlung dieser Auffälligkeiten in Kenntnis gesetzt.

7.2 Sicherstellungen der Nachbeobachtung von Versuchspersonen nach unerwünschten Ereignissen

Patienten nach unerwünschten Ereignissen, die im Rahmen der Studie aufgetreten sind, werden in der Sprechstunde des Prüfers nachbetreut.

8. Statistik

8.1 Definition des primären Endpunktes und der sekundären Endpunkte

Der primäre Endpunkt der Studie ist die Veränderung des Muskelmetabolismus zwischen Tag 1 und Woche 16

Die sekundären Endpunkte sind:

1. Die Veränderung der klinisch gemessenen Muskelkraft zwischen Tag 1 und Woche 16.
2. Die Veränderung des Fettgehalts der Muskulatur zwischen Tag 1 und Woche 16.

3. Die Veränderung des Muskelmetabolismus zwischen Tag 1 und Woche 16.
4. Die Veränderung der laborchemischen Parameter zwischen Tag 1 und Woche 16.

8.2 Geplante Anzahl Versuchspersonen mit nachvollziehbarer Begründung

5-6 DMD Patienten werden für diese Pilotstudie benötigt. Eine Poweranalyse ist nicht möglich, da es sich um eine Pilotstudie handelt. Die nachvollziehbare Begründung für die gewählte Anzahl Versuchspersonen sind ethische Überlegungen. Wir erwarten einen positiven Trend, der sich auch bei dieser kleinen Anzahl Patienten eine Poweranalyse für Crossoverstudien ermöglicht. Die geplante Studie ist eine Pilotstudie, deren Ziel es ist, durch eine breite Analyse von klinischen, bildgebenden, muskelbioptischen Untersuchungen den klinisch und statistisch besten Parameter für eine nachfolgende randomisierte Cross-over-Studien zu eruieren. Da noch keine Studien zur Anwendung von Metformin und L-Arginin publiziert worden sind, wurde diese Pilotstudie geplant, um Parameter für künftige Poweranalysen zu etablieren. Die geplante Patientenzahl basiert auf der bekannten Zahl an betroffenen Patienten, welche die Einschlusskriterien erfüllen. Diese Zahl ist ausreichend, um Trends aufzuzeigen und die Anzahl in weitere Studien einzuschliessende Patienten zu planen.

8.3 Beschreibung der vorgesehenen statistischen Methoden und der geplanten Zwischenauswertung

Nicht parametrische Analyse mittels Wilcoxon-Mann Test.

8.4 Geplantes Signifikanzniveau

p<0..05

8.5 Umgang mit fehlenden Daten sowie Daten bei vorzeitigem Studienabbruch von Teilnehmern

Last observation carried forward

8.6 Definition der Auswertungsgruppen

ITT Analyse

9. Studienspezifische Vorsichtsmassnahmen und Pflichten

9.1 Studienspezifische Vorsichtsmassnahmen

Studienspezifischen Vorsichtsmassnahmen sind keine notwendig.

9.2 Abschlussuntersuchung bei vorzeitigem Rücktritt aus der Studie

In diesem Fall ist zur Sicherheit des Kindes eine medizinische Abschlussuntersuchung notwendig. Die Untersuchung umfasst eine klinische Untersuchung, wie auch die Messung von Vitalparametern (Blutdruck, Puls) und eine Laborkontrolle. Es werden Kreatinkinase, L-Arginin, die Nüchtern-Glucose, das Nüchtern-Insulin, das Hba1c, HDL, LDL, Triglyceride, Adiponectin, Leptin, eine arterielle Blutgasanalyse, Laktat, Laktat-Pyruvat-Quotient), die Blutchemie mit Leber- und Nierenwerten (GOT GPT, Kreatinin, Harnstoff) und den Elektrolyten und das Differentialblutbild, bestehend aus Blutplättchen, den roten und weissen Blutkörperchen, bestimmt.

10. Pflichten des Prüfers

Die Studie wird gemäss Protokoll, GCP und den geltenden gesetzlichen Bestimmungen durchgeführt. Der Prüfer nimmt die Pflichten des Prüfers und des Sponsors gemäss GCP wahr. Der Prüfer bestätigt, dass er diese kennt und wahrnimmt.
Schwerwiegende unerwünschte Ereignisse, Protokolländerungen, sowie ein Zwischen- und Abschlussbericht werden Swissmedic und der Ethikkommission zugestellt.
Das Universitätskinderspital beider Basel (UKBB) ersetzt den Patienten Schäden, welche gegebenenfalls im Rahmen des klinischen Versuchs auftreten. Zu diesem Zweck hat das Universitätskinderspital beider Basel (UKBB) zu Gunsten der Patienten eine Versicherung bei der Versicherung Rimas Insurance-Broker AG, Leonhardsstrasse 55, 4051 Basel abgeschlossen.
Stellt der Patient oder seine Eltern während oder nach dem klinischen Versuch gesundheitliche Probleme oder andere Schäden fest, so soll(en) er / sie sich an den verantwortlichen Arzt / die verantwortliche Ärztin (PD Dr.med. Dirk Fischer / Dr.med. Patricia Hafner) wenden. Er / Sie wird die notwendigen Schritte einleiten.

11. Ethische Überlegungen

11.1 Bewertung des Risiko-Nutzen Verhältnisses

Die Muskeldystrophie vom Typ Duchenne ist eine schwerwiegende progrediente neuromuskuläre Erkrankung mit bislang nur geringen symptomatischen Therapiemöglichkeiten. Betroffene Patienten versterben meist zwischen dem 20. und 30. Lebensjahr aufgrund einer generalisierten Muskelschwäche, meist an kardiorespiratorischen Komplikationen. Die infauste Prognose rechtfertigt unseres Erachtens die breite klinische, bildgebende und semiinvasive Untersuchung des Muskelmetabolismus in vitro an einer kleinen Studienpopulation.

11.2 Beschreibung, warum besonders schützenswerte Versuchspersonen eingeschlossen werden

Die Duchenne Erkrankung manifestiert sich ab dem Kleinkindesalter und eine frühzeitige Therapie kann schwerwiegende Komplikationen kardiovaskulärer wie auch respiratorischer Art verzögern und somit die Lebensqualität länger erhalten.

Um die Wirkung von Metformin und L-Arginin auf den Muskel bei Knaben mit Muskeldystrophie Dychenne valide zu untersuchen, ist es unumgänglich besonders schützenswerte Versuchspersonen (Knaben im Alter von 7-10 Jahren) einzuschliessen. In diesem Alter sind Veränderungen am Muskel erkennbar, Muskelmasse ist jedoch in weiten Teilen erhalten, histologisch beurteilbar und funktionell. Im späteren Alter sind alle Patienten rollstuhlpflichtig, weshalb die (verbleibenden) klinischen Fähigkeiten dann nur noch eingeschränkt beurteilbar sind. Aufgrund der bereits stark ausgeprägten fettigen Umbauvorgänge im Muskel sind dann eine klinische, bildgebende und histologische Beurteilung sowie eine therapeutische Modifikation des Energiemetabolismus nicht mehr sinnvoll.

11.3 Andere ethische Aspekte

Die Teilnahme des Patienten an dieser Studie ist freiwillig. Bei Verzicht auf die Teilnahme haben die Eltern und der Patient keine Nachteile für die weitere medizinische Betreuung zu erwarten. Das gleiche gilt, wenn die Eltern und der Patient ihre dazu gegebene Einwilligung zu einem späteren Zeitpunkt widerrufen. Diese Möglichkeit haben sie jederzeit. Einen allfälligen Widerruf ihrer Einwilligung beziehungsweise den Rücktritt von der Studie müssen nicht begründen werden. Im Falle eines Widerrufes werden die bis zu diesem Zeitpunkt erhobenen Daten weiter verwendet und die im Rahmen der Studie erhobenen Proben (Gewebe, Blut) vernichtet. Falls sie ihre Einwilligung widerrufen, wird der Patient zu seiner Sicherheit abschliessend medizinisch untersucht.

Sollte sich die Studienhypothese bewahrheiten, d.h. die Wirksamkeit von Metformin und L-Arginin bei guter Verträglichkeit nachgewiesen werden, würden alle Studienteilnehmer direkt von der Medikation profitieren. Zudem wäre es dann unser Ziel, eine grössere Zahl an Probanden in einer Crossover-Studie zu untersuchen.

12. Qualitätskontrolle und Qualitätssicherung: Beschreibung und Massnahmen

12.1 Qualitätskontrolle

Zur Gewährleistung der Qualität der Studiendurchführung und der Daten wird ein Monitoring der Studie, von einer studienunabhängigen Person durchgeführt. Es werden alle Einschluss- und Ausschlusskriterien kontrolliert, ob die Daten korrekt in das CRF eingetragen wurden, ob die Drug Accountability stimmt und ob während der Studie SAE’s aufgetreten sind.

12.2 Vertraulichkeit der Daten

In dieser Studie werden persönliche Patientendaten erfasst. Diese Daten werden anonymisiert. Sie sind nur Fachleuten zur wissenschaftlichen Auswertung zugänglich. Die zuständigen Fachleute des Sponsors (oder deren Beauftragte) können im Rahmen eines sog. Monitorings oder Audits die Durchführung der Studie überprüfen. Diese, sowie im Rahmen von Inspektionen auch die Mitglieder der zuständigen Behörden, können Einsicht in die Originaldaten nehmen. Ebenso kann die zuständige Ethikkommission Einsicht in die Originaldaten nehmen. Während der ganzen Studie und bei den erwähnten Kontrollen wird die Vertraulichkeit strikt gewahrt. Der Name der Patienten wird in keiner Weise in Rapporten oder Publikationen, die aus der Studie hervorgehen, veröffentlicht.

Die Papierdokumente werden nach Abschluss der Studie während der Dauer von 10 Jahren im Archiv des UKKB, in einem abschliessbaren Raum aufbewahrt. Die Blutproben und die histologischen Präparate der Patienten werden nach Abschluss der Studie oder vorzeitigem Rückzug gemäss den SAMW Richtlinien für Biobanken und den spitalinternen Richtlinien für die Vernichtung von Blutproben und histologischen Präparaten vernichtet. In der Krankenakte wird vom Prüfarzt vermerkt, dass der Patient an der Pilotstudie teilnimmt. Sämtliche Daten, welche im Rahmen der Studie erhoben werden, werden zuerst in die Krankenakte eingetragen. Von da aus erfolgt zu einem späteren Zeitpunkt der Eintrag in das CRF. Es werden keine Daten direkt in das CRF eingetragen.

13. Verfahren zur Drug Accountability

Anlässlich der Visite 1, 3 und 4 werden dem Patienten L-Arginin, in den durch die Spitalapotheke vorbereiteten Kapseln, sowie 50 Tabletten Metformin (Metfin®) à 250 mg abgegeben. Der Patient wird gebeten anlässlich der Visiten 2, 3, 4, 5 und 6 die Studienmedikamente, sowie auch die leeren Blister und Schachteln mitzunehmen. Ein Mitarbeiter des Studienteams zählt und bilanziert die zurückgebrachten Tabletten und kann somit die korrekte Einnahme überprüfen. Er trägt diese in einem speziell hierzu kreierten Formular ein.

14. Verfahren zur Erfasssung der Compliance

Der Prüfer berechnet die Compliance anlässlich jeder Visite mittels folgender Formel berechnet: Die Anzahl der eingenommen Filmtabletten wird durch die Anzahl der verschriebenen Filmtabletten dividiert und mit 100 multipliziert. Das Resultat ergibt eine Zahl in Prozenten.

Anlässlich des Monitorings wird durch eine qualifizierte Person die Anzahl verabreichten/eingenommenen Studienmedikamente kontrolliert.

15. Vorgesehene Beschriftung der Prüfpräparate

Die Prüfpräparate werden mit Etiketten versehen. Folgende Angaben werden enthalten sein: Studiennummer, Name des Prüfers, für klinischen Versuch, Lotnummer, Verfalldatum, Lagerungsbedingungen, Patientennummer, für Kinder unzugänglich aufbewahren.

16. Publikation der Resultate

Die Resultate der Studie werden unabhängig vom Ergebnis öffentlich zugänglich gemacht. Diese werden in einem medizinischen Journal publiziert.

17. Referenzen

1 Merry TL et al. Downstream mechanism of nitric oxide-mediated skeletal muscle glucose uptake during contraction. *Am J Physiol Regul Integr Comp Physiol.* 2010, Dec;299(6):R1656-653McGee SL

2 Arzneimittelkompendium der Schweiz 2011

3 Yanovski JA et al. Effects of metformin on body weight and body composition in obese insulin-resistant children: a randomized clinical trial. Diabetes, 2011 Feb;60(2):477-85

4 Schwedhelm E et al. Pharmacokinetic and pharmacodynamic properties of oral L-citrulline and L-arginine: impact on nitric oxide metabolism. Br J Clin Pharmacol. 2008 Jan;65(1):51-9

5 Sperl W et al. resolution respirometry of permeabilized skeletal muscle fibers in the diagnosis of neuromuscular disorders. *Mol Cell Biochem* 1997; 174:71-8

6 Kuznetsov AV et al. Impaired mitochondrial oxidative phosphorylation in skeletal muscle of the dystrophin-deficient mdx mouse. *Mol Cell Biochem*. 1998; 183:87-96

7 Millay DP et al. Genetic and pharmacologic inhibition of mitochondrial-dependent necrosis attenuates muscular dystrophy. *Nat Med.* 2008; 14:442-7

8 Brenman J.E. et al. Nitric oxide synthase complexed with dystrophin ans absent from skeletal muscle sarcolemma in Duchenne muscular dystrophy *Cell*. 1995; 82:743–752

9 http://microarray.cnmcresearch.org/pga

10 Merry TL et al. Downstream mechanism of nitric oxide-mediated skeletal muscle glucose uptake during contraction. *Am J Physiol Regul Integr Comp Physiol.* 2010, Dec;299(6):R1656-653McGee SL,

11 McConell GK et al. Central role of nitric oxide synthase in AICAR and caffeine-induced mitochondrial biogenesis in L6 myocytes. *Appl Physiol.* 2010 Mar;108(3):589-95

12 Hargreaves M. AMPK-mediated regulation of transcription in skeletal muscle *Clin Sci (Lond).* 2010 Jan 26;118(8):507-18

13 Mizunoya W et al. Nitric-oxide donors improve prednisone effects on muscular dystrophy in the mdx mouse diaphragm. *Am J Physiol Cell Physiol* 2011

14 Archer JD et al. Persistent and improved functional gain in mdx dystrophic mice after treatment with L-arginine and deflazacort. *FASEB J.* 2006 Apr;20(6):738-40

15 Wehling-Henricks et al. Loss of positive allosteric interactions between neuronal nitric oxide synthase and phosphofructokinase contributes to defects in glycolysis and increased fatigability in muscular dystrophy *Hum Mol Genet*. 2009 September 15; 18(18): 3439–3451

16 Kim YW et al. Metformin restores the penile expression of nitric oxide synthase in high-fat-fed obese rats *J Androl* 2007;28:555-560

17 Schwedhelm E. et al. Pharmacokinetic and pharmacodynamic properties of oral L-citrulline and L-arginine: impact on nitric oxide metabolism_Br J Clin Pharmacol 2008 Jan;65(1):51-9. Epub 2007 Jul

18 Smith H et al. Nitric oxide precursors and congenital heart surgery: A randomized controlled trial of oral citrulline J Thorac Cardiovasc Surg. 2006 Jul;132(1):58-65

19 Bennett-Richards et al. Oral L-arginine does not improve endothelial dysfunction in children with chronic renal failure. Kidney Int. 2002 Oct ;62(4) :1372-8

20 Koga et al. MELAS an L-arginine therapy: pathophysiological therapy of stroke-like episodes. Ann N Y Acad Sci. 2010 Jul;1201:104-10.
