# Supplementary material for: Improved Muscle Function in Duchenne Muscular Dystrophy through L-Arginine and Metformin: An Investigator-Initiated, Open-Label, Single-Center, Proof-Of-Concept-Study
Source: PLoS One. 2016 Jan 22;11(1):e0147634. doi: 10.1371/journal.pone.0147634 (PMC4723144; doi:10.1371/journal.pone.0147634)
Supplement: S3 Appendix — (PDF) [file pone.0147634.s003.pdf]

RESEARCH PROJECT – PROJECT PLAN

1. Titel of the research project

Pilot study to investigate the effectiveness of L-arginine and metformin in seven to ten year old children with Duchenne muscular dystrophy

Version 4, 19th of April 2012

Investigator:

Name/Title: Dirk Fischer, MD

Departement: Neuropaediatrics

Hospital: University of Basel Children's Hospital

Signature:…………………………… Date: 19.04.2012

Co-Investigator:

Patricia Hafner, MD, resident physician Neuropediatrics, University of Basel Children's Hospital

List of staff members:

Prof. Peter Weber, MD, PhD, head of Neuropediatrics, University of Basel Children's Hospital

Urs Pohlman, MD, resident physician Neuropediatrics, University of Basel Children's Hospital

Arne Fischman, MD, senior physician neuroradiology, University of Basel Children's Hospital

2. Background of medical products

2.1 Description and indication of the investigated compounds

L-arginine

L-arginine is a half-essential amino acid that can be synthesized by the human organism. However, the produced amount is not sufficient to satisfy demand particularly in children and adolescents. L-arginine is essential for this age group and has to be ingested by food. It is included in relevant concentrations in nuts, seeds as well as in animal products such as pork meet or salmon. Based on a daily protein intake of approximately 70-90g, an arginine supply of 2-5g per day is usually achieved.

L-arginine is an important metabolite of the urea cycle. As exclusive precursor it’s here metabolized to nitric oxide (NO) by the nitric oxide synthase (NOS). An augmentation of plasma L-arginine is leading to an increased production of NO, which stimulates the formation and function of mitochondria in the skeletal muscle (1). L-arginine is an authorized food additive and is therefore not regulated by Swissmedic

Metformin (Metfin®)

Metformin (Metfin®) is an oral biguanid antidiabetic drug. It’s approved for the treatment of increased insulin resistance and increased blood glucose levels by Swissmedic. The goal of decreasing blood glucose levels is primary achieved by decreasing insulin resistanc in liver and muscle cells. Only in presence of insulin the basal as well as the postprandial plasma glucose concentration is depressed. Metformin has no stimulating effect on the insulin secretion and is not triggering hypoglycemia(2). In the European Union and in Switzerland metformin is approved for this indication from the age of 10 years in children and adults. The safety and efficacy of the application of metformin in children and adolescents was reviewed before approval in a randomized, double blind study on 82 children - aged 10 to16 years. Newest data on 53 insulin resistant children aged 6 to12 years (including 19 children aged 6 to10 years) showed good tolerance (3). There were no serious adverse reactions in the applied dose of 2000mg metformin daily.

2.2 Dose selection, scheme of dosing and treatment period

L-arginine

The normal L-arginine plasma concentration in 8 to 11 years old children lies between 54.29 and 115.33µmol/l. In this study we are aiming to double the plasma concentration (minimum 100µmol/l –maximum 200µmol/l). Using an oral application of 3 x2.5g respectively 3 x 5g/ m^2 body surface/ day Bennett et al. (4) achieved a doubling of L-arginine plasma concentration in children aged 7 to 17 years. This is in line with the dose of 3 x 0.3-0.5mg/kg body weight/d

used by Koga et al. (5) to augment L-arginine plasma concentration > 100µmol/l in patients with MELAS. Therefore in our study a dose of 3 x 2.5 g L-arginine daily over a time period of 16 weeks is administered.

Metformin (Metfin®)

Unpublished own data show insulin resistance in boys with Duchenne muscular dystrophy. For the planned study a dose of metformin 2 x 250mg/d for 16 weeks is planned. The rationale for the dose selection is to achieve a sufficient activation of muscle metabolism with the lowest risk of gastrointestinal side effects. At the beginning of medication with metformin nausea, vomiting, diarrhea and stomach pain can occur. These symptoms often resolve spontaneously.

3. Aim and purpose

3.1 Background, rationale and aim of the study

Duchenne Muscular Dystrophy (DMD) is an X-linked recessive neuromuscular disorder that affects 1 in 3.500-6.000 male births. Although the disease causing gene product, dystrophin, is present in many different tissues throughout the body, the disease pathology is restricted predominantly to muscle tissue. In the muscle, dystrophin is located close to the inner surface of the plasmalemma and interacts as a structural protein both with a number of membrane proteins that form the dystrophin-associated glycoprotein complex (DGC) and cytoskeletal proteins (6),(7). Therefore, loss of dystrophin in DMD is associated with a loss of cytoskeletal and sarcolemmal integrity. Destroyed muscle fibers are rapidly and irreversibly replaced by connective and fatty tissue. Clinically this leads to a delayed motor development at the age of 3 to 4 years. Later in the course the muscle weakness increases and free ambulation is lost around the age of 10 to 12 years. Scoliosis, respiratory disorders and cardiomyopathy are common problems. The life span is improved substantially amongst others because of non-invasive ventilation.

It is believed that this structural defect gives rise to dysregulated calcium homeostasis through mechano-sensitive Ca^{++}-channels, activation of proteases, such as calpain, and increased production of reactive oxygen species (ROS), which cause protein and membrane damage. One of the major sources of cellular ROS are mitochondria, implying altered mitochondrial function in DMD. However, while patients with mitochondrial dysfunction disorders frequently display impaired muscle function (8), mitochondrial dysfunction as a feature of DMD is not generally accepted despite numerous reports. One of the first publications that described impaired oxidative phosphorylation as a feature of DMD was reported 1985 (9). Later, using ^{31}P magnetic resonance spectroscopy, increased ADP and Pi levels relative to ATP and reduced phosphocreatine levels were found in muscle of DMD

patients (10). Sperl et al. (11) also reported decreased oxidation rates in muscle biopsies from DMD patients and some indication of loose coupling of oxidative phosphorylation in mitochondria from those patients. These findings were also supported by later observations of reduced rates of respiration and lower activities of enzymes of the mitochondrial respiratory chain in biopsy samples of a DMD patient (12).

Some of this mitochondrial dysfunction is recapitulated in the *mdx*-mouse model of DMD. Analysis of skeletal *mdx* muscle showed a 50% decrease in the activity of all respiratory chain linked enzymes compared to control animals (12). The authors also reported that isolated mitochondria from *mdx* muscles had only 60% of maximal respiration rates compared to control and attributed this impairment to a Ca^{++}-overload of dystrophin-deficient muscle fibers. Interestingly, this study identified no deficiencies in the cardiac muscle. Contrary to that, Braun et al. (2001) (13) reported that irrespective of muscle type, the absence of dystrophin had no effect on the maximal capacity of oxidative phosphorylation, or on coupling between oxidation and phosphorylation. However, in the myocardium and *m. soleus*, the coupling of mitochondrial creatine kinase to adenine nucleotide translocase was attenuated as evidenced by the decreased effect of creatine on the Km for ADP in the reactions of oxidative phosphorylation. Finally, Millay et al. (14) reported a strong link between mitochondrial-dependent necrosis and muscular dystrophy in several mouse models (incl. the *mdx*-model), which strongly suggests that mitochondria play a major role in the pathology of DMD. Consistent with an impaired mitochondrial function in DMD, a low fat utilization as energy substrate in the early stages of the disease has been suggested (15, 16). This hypothesis is supported by observations that muscle tissue is increasingly being replaced by fatty tissue in DMD patients (17).

In DMD, loss of dystrophin also results in a severe reduction of neuronal nitric oxide (NO) synthase (nNOS) activity (18), which under normal conditions converts intramuscular L-arginine to nitric oxide (NO) (19) (Fig 1a, b). An important physiological role of NO is to stimulate mitochondrial biogenesis by increasing the concentration SIRT1 and PGC-1α (20), NO is also critical for regulating muscular energy balance also through activation of AMP-activated protein kinase (AMPK) (21). It is thought that NO and AMPK both increase mitochondrial function and biogenesis through independent mechanisms in a synergistic manner (Fig 1c). Therefore, impaired nNOS function could contribute to the observed mitochondrial dysfunction in DMD. Increasing NO levels to stimulate mitochondrial function, to reduce oxidative stress, and to improve fat utilization for energy production appeared a promising approach to us to ameliorate the pathology of DMD. This study therefore aimed to evaluate the subclinical and clinical benefits of the NO precursor L-arginine, and the pharmacological AMPK activator metformin, in DMD.

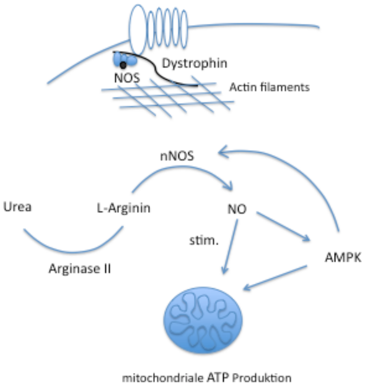

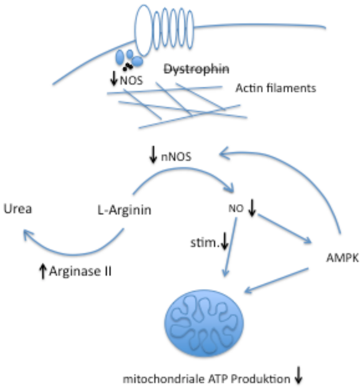

a) healthy muscle cell metabolism

b) impaired muscle cell metabolism in Duchenne muscular dystrophy

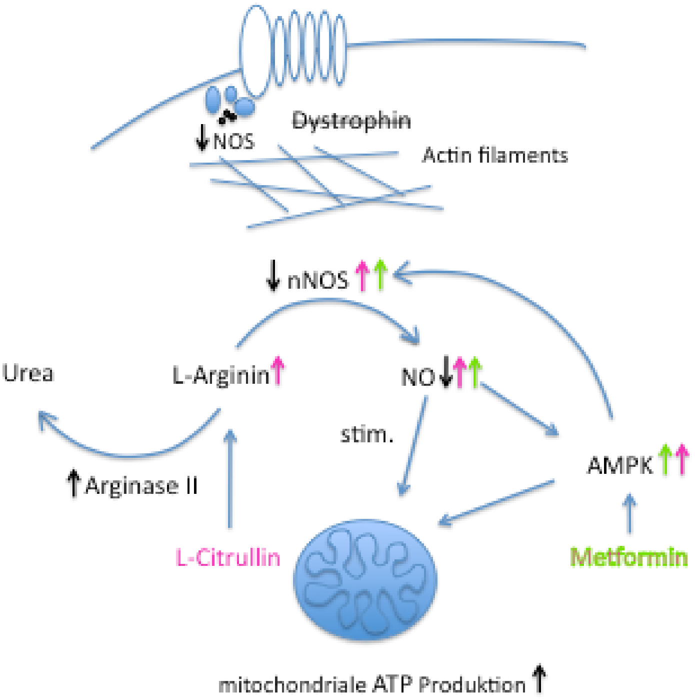

c) muscle cell metabolism in DMD treated with L-arginine and metfomin

3.2 Reason to conduct the study, study population

This study investigates if a combined treatment with L-arginine and metformin leads to an improvement of muscle energy metabolism in patients with Duchenne muscular dystrophy and by this to an improvement of muscle force, reduction of early fatigability and to a deceleration of muscle wasting.

The efficacy of a treatment with L-arginine and metformin will be tested in a small pilot trial in 5 to 6 ambulatory DMD patients aged 7 to 10 using by clinical, imaging an immunohistochemical methods as outcome measures.

3.3 Hypothesis

Elevated intramucuslar nitric oxide (NO) production should lead to an improvement of muscle force, a deceleration of muscle wasting and to prolonged free ambulation in DMD

4. Study design

4.1 Primary and secondary outcomes

Primary outcomes

In vivo measurement of muscle metabolism

Lean body mass is measured with dual-energy X-ray absorptiometry. This is a non-invasive method that allows radiographing patients with weakly dosed X-radiation. Fat, muscle and water attenuates the radiation differently. This attenuation allows calculating the body composition precisely. The calculation of the muscular energy metabolism is effected by calorimetry.

Secondary outcomes

1. In vitro measurement of muscle metabolism

Muscle MRI is a promising method to measure the effectiveness of therapeutic interventions on deceleration of the fatty degeneration of muscle tissue. The examination with MRI has several advantages: it's not invasive, fast and can picture the relevant muscular structures ideally. With specially developed sequences (Dixon method) fatty degeneration can be measured and the further course can be assessed. It will take 20 minutes to perform the MRI sequences. No contrast agent is administered. The examination is not performed under anesthetic.

The examination is performed in collaboration with the Departement of Radiology, University of Basel Hospital and is performed at Baseline as well as after 16 week of treatment.

2. In vitro measurement of the fatty degeneration of the muscle by MRI

At the beginning of the study as well as after treatment period of 16 weeks a muscle punch biopsy of vastus lateralis muscle is performed. The procedure is performed in general anesthesia by an experienced pediatric surgeon. The histopathological examination includes NO analysis and will be conducted at the Department of Neuropathology, University of Basel.

3. Clinical measurement of muscle force

The motor function measure is a validated test to measure the muscle force in ambulatory und non-ambulatory DMD patients. This test serves as a progression parameter to determine the advancement of muscle weakness. Additionally to this standardizes measure the force for fist closure, extension and flexion of the elbow, hip flexion and hip extension as well as foot elevation is measured by myometry within a physiotherapeutic examination.

4. Laboratory parameters

Differential blood count	
Chemistry	transaminases, creatinine, electrolytes, urea
Marker of muscle necrosis	creatine kinase
Amino acids	L-citrulline, L-arginine
Marker of glucose metabolism	fasting glucose, fasting insulin, HbA1c
Marker of fat metabolism	HDL, LDL, triglycerides, adiponectin, leptine
To exclude a lactic acidosis	blood gas analysis, lactate level, lactate-pyruvate-quotient

4.2 Study design

This study is a pilot study. The study design is shown as a flowchart in table x. 5 to 6 children aged between 7 and 10 years should be enrolled into the study and treated over a time period of 16 weeks between October 2011 and January 2012.

**Screening**	**Visit 1 / Day 1**	**Visit 2 / week 2**	**Visit 3 / Week 4**	**Visit 4 / Week 8**	**Visit 5 / Week 16**
Patient Information	physical examination	physical examination	physical examination	physical examination	physical examination
Informed Consent	vital signs	vital signs	vital signs	vital signs	vital signs
Inclusion Exclusion Criteria	blood withdrawal	blood withdrawal	blood withdrawal	blood withdrawal	blood withdrawal
	DEXA / calorimetry				DEXA / calorimetry
	muscle function				muscle function
	MRI				MRI
	muscle biopsy				muscle biopsy
	distribution study drug				
	distribution diary	diary check	diary check	diary check	check and collect diary

Screening

The inclusion and exclusion criteria are verified. If the criteria are met and the patient still wishes to participate in the study the patient and the parents are again informed about the

study content. The patient will declare his assent while the caregivers will sign the informed consent form. The patient will in this case be enrolled into the study.

Visit 1: One day hospitalization

History will be taken and clinical examination is performed by the investigator. The study nurse then measures blood pressure and pulse. A blood sample is taken while the amount of blood is about 15ml (around 3 tablespoons). Marker of muscle necrosis, kreatine kinase, the amino acids L-arginine and L-citrulline, marker of glucose metabolism (fasting glucose, fasting insulin, HbA1c), marker of fat metabolism (HDL, LDL, triglycerides, adiponectin, leptine), differential blood count and chemistry (transaminases, creatinine, electrolytes, urea) are determined.

Thereafter DEXA is performed to calculate the portion of fat and muscle content of the body. This is a non-invasive method that allows radiographing patients with weakly dosed X-radiation. Fat, muscle and water attenuates the radiation differently. This attenuation allows calculating the body composition precisely. The calculation of the muscular energy metabolism is effected by calorimetry.

A trained physiotherapist is examining the patient's muscle function using an international validated motor function test (motor function measure) as well as timed motor tests. Then an MRI of the leg muscles is performed to reliably determine the amount of fat tissue. The time needed for the thigh and calf muscles is about 3 minutes each. In this two times 3 minutes the patients have to keep their legs quite to allow evaluation of the images. Because of the short examination time we perform this examination without sedation or anesthesia. The total examination time in the MRI with bedding takes about 20 minutes. Parents can accompany their children next to the MRI machine. As a last examination the muscle punch biopsy is performed. Therefore a little piece of muscle from vastus lateralis muscle has to be taken. The procedure is performed in general anesthesia by an experienced pediatric surgeon and takes about 20minutes. The medication for the whole study period is dispensed.

The day after the participant can leave the hospital.

Visit 2: (+/- 3 days ambulatory or by phone call for patients who live more than 500km away from the study center)

The history is taken and the clinical examination is performed by the investigator or the delegated pediatrician near the patient's home. The study nurse then measures blood pressure and pulse. A blood sample is taken while the amount of blood is about 15ml (around 3 tablespoons). Marker of muscle necrosis, kreatine kinase, the amino acids L-arginine and L-citrulline, marker of glucose metabolism (fasting glucose, fasting insulin,

HbA1c), marker of fat metabolism (HDL, LDL, triglycerides, adiponectin, leptine), differential blood count, chemistry (transaminases, creatinine, electrolytes, urea) are determined.
In case that the blood withdraw is performed at the place of residence of the patient abroad blood sample is sent to Basel for examination.

Visit 3: (+/- 3 days ambulatory or by phone call for patients who live more than 500km away from the study center)
The history is taken and the clinical examination is performed by the investigator or the delegated pediatrician near the patient’s home. The study nurse then measures blood pressure and pulse. A blood sample is taken while the amount of blood is about 15ml (around 3 tablespoons). Marker of muscle necrosis, kreatine kinase, the amino acids L-arginine and L-citrulline, marker of glucose metabolism (fasting glucose, fasting insulin, HbA1c), marker of fat metabolism (HDL, LDL, triglycerides, adiponectin, leptine), differential blood count, chemistry (transaminases, creatinine, electrolytes, urea) are determined.
In case that the blood withdraw is performed at the place of residence of the patient abroad blood sample is sent to Basel for examination.

Visit 4: (+/- 3 days ambulatory or by phone call for patients who live more than 500km away from the study center)
The history is taken and the clinical examination is performed by the investigator or the delegated pediatrician near the patient’s home. The study nurse then measures the blood pressure and the pulse. A blood sample is taken while the amount of blood is about 15ml (around 3 tablespoons). Marker of muscle necrosis, kreatine kinase, the amino acids L-arginine and L-citrulline, marker of glucose metabolism (fasting glucose, fasting insulin, HbA1c), marker of fat metabolism (HDL, LDL, triglycerides, adiponectin, leptine), differential blood count, chemistry (transaminases, creatinine, electrolytes, urea) are determined.
In case that the blood withdraw is performed at the place of residence of the patient abroad blood sample is sent to Basel for examination.

Visit 5: (1/- 3 days, one day hospitalization)
The history is taken and the clinical examination is performed by the investigator. The study nurse then measures blood pressure and pulse. A blood sample is taken while the amount of blood is about 15ml (around 3 tablespoons). Marker of muscle necrosis, kreatine kinase, the amino acids L-arginine and L-citrulline, marker of glucose metabolism (fasting glucose, fasting insulin, HbA1c), marker of fat metabolism (HDL, LDL, triglycerides, adiponectin, leptine), differential blood count, chemistry (transaminases, creatinine, electrolytes, urea) are determined.

After that DEXA is performed to calculate the portion of fat and muscle content of the body. The calculation of the muscular energy metabolism is effected by calorimetry.
A physiotherapist is measuring muscle force of the patient (as described in visit 1).
Then an MRI of the legs is performed (as described in visit 1). As a last examination the muscle punch biopsy is performed (as described in visit 1). Know the study is finished for the patient and he can leave the hospital.

4.2.1 Exclusion of patients from the study

- Withdrawal of consent
- Serious Adverse Event
- Use of medication listed in the exclusion criteria section?
- Protocol violations caused by the patient (noncompliance)
- Logistical reasons (patient moves to another city, etc.)
- Circumstances which do not permit any more regular visits in the context of the study
- Abnormal laboratory values incl. liver or renal function tests (transaminases >2xULN, creatinine >2xULN)
- Increase in blood pressure >95 percentile
- Hyperlactacidemia (>4 mmol/l, pH<7.25)

4.3 Actions to minimize bias

Patients in the swiss DMD registry are screened in alphabetic order regarding in- and exclusion criteria. They are asked to participate in this study. There is neither randomization nor blinding. One investigator performs the study visits including case history and clinical examination. The physiotherapists assessing the motor function measure were trained and certificated in Lyon, France, where MFM was established and validated.

5. Selection of patients

5.1 Recruitment

The patients participating in this study will be recruited from the outpatient ward of the University of Basel Children’s Hospital as well as from the swiss registry and DMD-Registry. At the time of the study conduct 120 patients are registered in the swiss registry.

5.2 Inclusion criteria

- Patients with molecular diagnosis of DMD
- Patients 7-10 years of age at time of screening

- Ambulant at screening

5.3 Exclusion criteria

- Previous participation in another therapeutic study for DMD within the last 3 months
- Use of L-arginine, L-citrulline or metformin within the last 3 months
- Known hypersensitivity to L-arginine or metformin
- Other chronic disease or clinical relevant limitation of renal, liver, heart or lung function to discretion of investigator

6. Assessment of efficacy

1. Measure of muscle metabolism by DEXA and calorymetriy at baseline and week 16.
2. Measure of the degree of fatty degeneration by MRI at baseline and week 16.
3. Measure of muscle metabolism in by histological examination of a muscle biopsy at baseline and week 16.
4. Measure of muscle force by motor funcion measure assessed at baseline and week 16.

7. Safety

7.1 Safety parameter: methods and time points

At every visit a clinical examination and measurement of vital signs were performed. At Day 1 and Week 16 laboratory tests of the following parameters were performed:

Differential blood count, as during the intake of metformin in the context of the marketing authorization study isolated cases of leukopenia, thrombopenia and hemolytic anemia were found, chemistry (transaminases, creatinine, electrolytes, urea), marker od muscle necrosis (creatine kinase), amino acids (L-arginine), marker of glucose metabolism (fasting glucose, fasting insulin, HbA1c), marker of fat metabolism (HDL, LDL, triglycerides, adiponectin, leptine).

In case of clinical significant changes (elevation of creatinine and transaminases >200% of normal value, concentration of lactate >8mmol/l) the intake of metformin was stopped.

If pathological incidental findings were detected independent of the known muscle disease, the patients were immediately informed and according to the current medical knowledge and instructed regarding the possibilities of further diagnostic assessments, respectively treatment of these abnormalities.

7.2 Follow-up of patients after unexpected events

After unexpected events patients are followed-up urgently by the investigator.

8. Statistics

8.1 Definition of primary and secondary endpoints

The primary endpoint is the change of muscle metabolism between baseline and week 16.

The secondary endpoints are:

1. Change of muscle force between baseline and week 16.
2. Change of the degree of fatty degeneration between baseline and week 16.
3. Change of laboratory parameters between baseline and week 16.

8.2 Determination of sample size

The sample size of 5-6 DMD patients was defined by the principal investigator for this pilot study. A power analysis was not possible. The sample size was chosen according to practical clinical and ethical considerations. Therefore, a total of five patients is regarded to be sufficient to obtain useful results to plan further studies. We expect a positive trend large enough to allow, albeit the small number of patients, a power analysis for further studies. The planned study is a pilot study with a broad variety of outcome measures (clinical, muscle imaging and muscle biopsies) with the aim to determine the best clinical and statistical measure for a following randomized cross-over study. As at this time point no studies for the use of metformin and L-arginine had been published, this pilot study was planned to establish outcome measures allowing future power analyses.

8.3 Statistical and analytical plans

For the comparisons between baseline and follow-up measurements a two-sided t test is planned.

8.4 Level of significance

$p<0.05$

8.5 Handling of missing data

Last observation carried forward

8.6 Definition of investigated groups

ITT Analysis

9. Study specific precautions and dutys

9.1 Study specific precautions

Study specific precautions are not necessary.

9.2 Follow-up visit in case of untimely withdrawal from the study

For the safety of the child a final medical exam is necessary. This exam includes a clinical examination as well as the measure of vital parameters (blood pressure, pulse) and a laboratory test. Creatine kinase, L-arginine, fasting glucose, fasting insulin, HbA1c, HDL, LDL, triglycerides, adiponectin, leptine, , blood chemistry with liver and kidney values (ASAT, ALAT, creatinene, urea) and electrolytes as well as a blood count consisting of thrombocytes, red and white blood cells are measured.

10. Duties of the investigator

The study is conducted according to the protocol, GCP and the legal requirements. The investigator assumes the obligations of the investigator and sponsor according to GCP. The investigator confirms that he knows the obligations and that he administers them accordingly. Serious adverse events, changes of the protocol as well as interim- and final reports will be sent to Swissmedic and to the ethics committee.

The University of Basel Children's Hospital (UKBB) indemnifies damage that is, as the case may be, caused by this trial. For this reason the University of Basel Children's Hospital (UKBB) has taken out an insurance policy at Rimas Insurance-Broker AG, Leonhardstrasse 55, 4051 Basel.

If the patient states health damages meanwhile or after the study he should informe the investigator (PD Dr.med. Dirk Fischer) or sub-investigator (Dr. med. Patricia Hafner) who will initiated the necessary procedures for the patients and their caregivers.

11. Ethical considerations

11.1 Evaluation of the cost-benefit-risk balance

Duchenne muscular dystrophy (DMD) is a severe progressive neuromuscular disorder with limited therapeutic possibilities so far. In most of the cases affected patients die because of cardiorespiratory complications between the age of 20 and 30 years. The unfavorable prognosis justifies the broad clinical, radiological and semi-invasive examination of the muscle metabolism in vitro in a small study population.

11.2 Reason of enrollment of highly sensitive subjects

DMD manifests since early infancy and an early therapy is expected to reduce severe cardiovascular and respiratory complications and preserve life quality. To assess the efficacy of metformin and L-arginine on muscle tissue in children with Duchenne muscular Dystrophy it's necessary to enroll subjects highly sensitive to the treatment effects (children aged of 7 to 10 years). At this age changes in muscle tissue are visible, however muscle mass is preserved, histologically assessable and functionally. At a higher age the patients lose their

ability to walk and the ability to assess remaining muscular skills is limited. In addition, because of the advanced fatty degeneration clinical, radiological and histological assessments as well as therapeutic modifications of the energy metabolism are not useful anymore.

11.3 Other ethical considerations

The participation on this study is voluntary. If patients don't want to participate in the study they don't have to fear disadvantages in the further medical care. The same is the case if patients or caregivers resign to continue the study. This decision needs not to be justified. In case of retraction the collected personal data will be further used, the collected probes (tissue, blood) will be destroyed. In case of retraction the patient is finally examined.
If the hypothesis of this study is right patients would profit directly from the medication. Our goal is to examine more patients in a crossover study.

12. Quality assurance: Description of the measures

12.1 Quality assurance

To guarantee the quality of conduction of the study and data collection a monitoring will be performed by an independent party. The inclusion and exclusion criteria will be checked; the registration of data in the CRF, the drug accountability and the registration of SAE's will be controlled.

12.2 Confidentiality of data

In this study personal data is acquired. This data is anonymized. Only specialists who are interpreting data have access. The dedicated specialists of the sponsor can on in the event of an external monitoring or audit inspect the conduct of the study and can retain insight into original data. The local ethical committee can retain insight into original data as well. During the conduct of the study and at the occasion of an audit confidentiality is always guaranteed. The names of the patients will not be published in rapports or publications that emerge from this study.
Documents are archived over a time period of 10 years in a lockable room at the UKBB. The blood samples as well as muscle tissue for histological analysis is destroyed after closing of the study or in case of retraction of the study. The participation in the study is noted in the patients' health record. All data collected in this study is primarily noted in the patients'' health record before being transferred into the CRF.

13. Method for drug accountability

On the occasion of visit 1 and 4 L-arginine and metformin is administered to the patient. The patient is advised to bring study medication as well as blisters and boxes to the study center at visit 2, 3, 4, 5 and 6. A member of the study team counts the material and prepares a balance sheet.

14. Method to assess the compliance

Study medication is distributed to the subjects during the site visits. Patients return the used/unused study drug at the next on site visit. On occasion of a monitoring the count of taken medication will be checked.

15. Labelling of study drug

The study drug is scored with a label including study number, name of the investigator, lot number, date of expiry, storage conditions, patient number and the information “store out of the reach of children”.

16. Publication of the results

The results of the study will be published in a public journal independent of the nature of results.

17. References

1. Merry TL, Lynch GS, McConell GK. Downstream mechanisms of nitric oxide-mediated skeletal muscle glucose uptake during contraction. American journal of physiology Regulatory, integrative and comparative physiology. 2010;299(6):R1656-65.
2. Schweizerisches Arzneimittelkompendium
3. Yanovski JA, Krakoff J, Salaita CG, McDuffie JR, Kozlosky M, Sebring NG, et al. Effects of metformin on body weight and body composition in obese insulin-resistant children: a randomized clinical trial. Diabetes. 2011;60(2):477-85.
4. Bennett-Richards KJ, Kattenhorn M, Donald AE, Oakley GR, Varghese Z, Bruckdorfer KR, et al. Oral L-arginine does not improve endothelial dysfunction in children with chronic renal failure. Kidney international. 2002;62(4):1372-8.
5. Koga Y, Povalko N, Nishioka J, Katayama K, Kakimoto N, Matsuishi T. MELAS and L-arginine therapy: pathophysiology of stroke-like episodes. Annals of the New York Academy of Sciences. 2010;1201:104-10.
6. Yoshida M, Ozawa E. Glycoprotein complex anchoring dystrophin to sarcolemma. Journal of biochemistry. 1990;108(5):748-52.
7. Campbell KP, Kahl SD. Association of dystrophin and an integral membrane glycoprotein. Nature. 1989;338(6212):259-62.
8. Smeitink J, van den Heuvel L, DiMauro S. The genetics and pathology of oxidative phosphorylation. Nature reviews Genetics. 2001;2(5):342-52.
9. Scholte HR, Luyt-Houwen IE, Busch HF, Jennekens FG. Muscle mitochondria from patients with Duchenne muscular dystrophy have a normal beta oxidation, but an impaired oxidative phosphorylation. Neurology. 1985;35(9):1396-7.

10. Kemp GJ, Taylor DJ, Dunn JF, Frostick SP, Radda GK. Cellular energetics of dystrophic muscle. Journal of the neurological sciences. 1993;116(2):201-6.
11. Sperl W, Skladal D, Gnaiger E, Wyss M, Mayr U, Hager J, et al. High resolution respirometry of permeabilized skeletal muscle fibers in the diagnosis of neuromuscular disorders. Molecular and cellular biochemistry. 1997;174(1-2):71-8.
12. Kuznetsov AV, Winkler K, Wiedemann FR, von Bossanyi P, Dietzmann K, Kunz WS. Impaired mitochondrial oxidative phosphorylation in skeletal muscle of the dystrophin-deficient mdx mouse. Molecular and cellular biochemistry. 1998;183(1-2):87-96.
13. Braun U, Paju K, Eimre M, Seppet E, Orlova E, Kadaja L, et al. Lack of dystrophin is associated with altered integration of the mitochondria and ATPases in slow-twitch muscle cells of MDX mice. Biochimica et biophysica acta. 2001;1505(2-3):258-70.
14. Millay DP, Sargent MA, Osinska H, Baines CP, Barton ER, Vuagniaux G, et al. Genetic and pharmacologic inhibition of mitochondrial-dependent necrosis attenuates muscular dystrophy. Nature medicine. 2008;14(4):442-7.
15. Hankard R, Gottrand F, Turck D, Carpentier A, Romon M, Farriaux JP. Resting energy expenditure and energy substrate utilization in children with Duchenne muscular dystrophy. Pediatric research. 1996;40(1):29-33.
16. Gaeta M, Messina S, Mileto A, Vita GL, Ascenti G, Vinci S, et al. Muscle fat-fraction and mapping in Duchenne muscular dystrophy: evaluation of disease distribution and correlation with clinical assessments. Preliminary experience. Skeletal radiology. 2012;41(8):955-61.
17. Hollingsworth KG, Garrood P, Eagle M, Bushby K, Straub V. Magnetic resonance imaging in Duchenne muscular dystrophy: longitudinal assessment of natural history over 18 months. Muscle & nerve. 2013;48(4):586-8.
18. Brenman JE, Chao DS, Xia H, Aldape K, Bredt DS. Nitric oxide synthase complexed with dystrophin and absent from skeletal muscle sarcolemma in Duchenne muscular dystrophy. Cell. 1995;82(5):743-52.
19. MacAllister RJ, Whitley GS, Vallance P. Effects of guanidino and uremic compounds on nitric oxide pathways. Kidney international. 1994;45(3):737-42.
20. Nisoli E, Carruba MO. Nitric oxide and mitochondrial biogenesis. Journal of cell science. 2006;119(Pt 14):2855-62.
21. McGee SL, Hargreaves M. AMPK-mediated regulation of transcription in skeletal muscle. Clin Sci (Lond). 2010;118(8):507-18.
